# Supplementary material for: A phenomenological comparison of the effects of blue light, red light and radio waves on the escape speed of Caenorhabditis elegans and the rate of closure of Gerbera jamesonii petals
Source: PLoS One. 2026 Apr 1;21(4):e0343498. doi: 10.1371/journal.pone.0343498 (PMC13043045; doi:10.1371/journal.pone.0343498)
Supplement: S1 Protocol — Summary of the procedure we used to obtain the results on G. jamesonii presented in this work. (DOCX) [file pone.0343498.s001.docx]

**A phenomenological comparison of the effects of blue light, red light and radio waves on the escape speed of *Caenorhabditis elegans* and the rate of closure of *Gerbera jamesonii* petals**

Alexander W. Kline^1¶^, Charles S. Beattie^1¶^, Addison K. Shenk^2&^, Samuel I. Spicher^2&^, Timothy A. Bloss^3^, Laura Tipton^3,4^, Marquis T. Walker^3^, Laura G. Vallier^5^, Kristopher L. Schmidt^2^*, and Giovanna Scarel^1^*

^1^Department of Physics and Astronomy, James Madison University, Harrisonburg, VA, United States of America

^2^Department of Biology and Chemistry, Eastern Mennonite University, Harrisonburg, VA, United States of America

^3^Department of Biology, James Madison University, Harrisonburg, VA, United States of America

^4^Department of Mathematics & Statistics, James Madison University, Harrisonburg, VA, United States of America

^5^Department of Biology, Hofstra University, Hempstead, NY, United States of America

*Corresponding Authors

E-mail: [scarelgx@jmu.edu](mailto:scarelgx@jmu.edu) (GS), [kristopher.schmidt@emu.edu](mailto:kristopher.schmidt@emu.edu) (KS)

^¶^ These authors contributed equally to this work

^&^ These authors also contributed equally to this work

**Short title**: Comparison of the effects of light and radio waves on biological organisms

**Protocol for *G. Jamesonii* experiments**

The results reported in the Main Text were produced by experiments we performed with *G. jamesonii* according to the protocol outlined below.

**Plant purchase**: three plants of *G. jamesonii* were purchased from Monrovia Inc. for each experiment set. Each plant consisted of one to five flowers. We selected plants with at least one bud close to blooming and flowering.

**Plants color**: flowers were yellow, red, pink, orange. Color was not counted as an experimental variable as it was not found to produce a significant difference in the rate of closure.

**Plants utilization**: in each experiment set, one plant was used as a control in dark, one as a control in solar white light and one as a sample in one of the illumination environments (either blue light, red light, or radio waves).

**Plants maintenance**: plants were provided with about 10 mL of water each day.

**Plants recycle**: one or two flowers per plant were utilized for one experiment. A plant would be used for a second experiment only in the case it developed a useful bud at least two weeks after being utilized for the previous experiment. After use, plants were donated.

**Plant and flower labels**: each plant was labeled with a progressive number N. The selected flower with useful bud was identified with an upper-case letter in alphabetical order and a lower-case letter indicating the environment in which the flower was placed (d for darkness, b for blue light, r for red light, 4xr for radio waves, and w for solar white light). For example, 55-A-d labeled the 55^th^ plant we purchased utilized in darkness for the first experiment. The presented labels were used in the data presented in S1 Dataset.

**Plants count**: The data presented in this research were obtained with a total of 18 plants from N=31 to 55 (N is the number defined above at the progressive number indicating the order in time in which the plant was purchased).

**Plants shielding**: during the experiments, plants tested in darkness, blue light, red light, and radio waves were kept in expanded polystyrene (EPS) enclosures to ensure shielding, as explained in section 1 of S1 Text.

**Plant’ location**: the plants tested in the EPS enclosures were in the laboratory kept in dark at about 17.5°C-18.5°C and with lights off. The flowers examined under exposure to solar white light were kept on a windowsill facing East (87° E) in a room kept at about 21°C and illuminated by sunlight in the morning. The photographs of *G. jamesonii* in solar white light were taken without room’s overhead lights on.

**Plant’ illumination**: plants tested in darkness were kept in EPS enclosures with no source of electromagnetic wave. Plants tested in blue and red light were kept in EPS enclosures with two light emitting diodes (LEDs- ALLECIN-3mm). The two LEDs faced the *G. jamesonii* flower selected for the rate of petal’s closure measurement. Each LED was connected through cables to a power supply (BK Precision 1665) set at 2.7 V for blue light and 1.8 V for red light. The LEDs illuminated the plants for the entire duration of the experiment. Plants tested in radio waves were kept in EPS enclosures with four radio devices (Sony ICF38 portable AM/FM) set at 100 MHz frequency and zero volume (i.e. no sound). The EPS enclosures were in a laboratory in which the overhead lights were off except for 1-2 minutes when photorecording. Plants tested in solar white light were kept on a windowsill facing East (87° E) of a room illuminated by sunlight in the morning. The room’s overhead lights were off during photorecording.

**P* intensity on plants**: The blue light had wavelength λ=450 nm and frequency ν=0.667 PHz (or 10^15^ Hz). Two blue LEDs generated a voltage of ΔV=0.15 mV when illuminating the whole surface of a 3TECBT photodetector with capacitance C=1503.4 pF and size 2.792 cm x 2.792 cm. Thus, light’s intensity was P=$\frac{1}{2}$Cν${\Delta V}^{2}$=12.0 mW [1]. Since the photodetector’s size matched that of the *G. jamesonii* petals, the specific intensity P* on the petals was estimated about 12 mW. The red light had wavelength λ=700 nm and frequency ν=0.429 PHz. Two red LEDs generated a voltage of ΔV=0.107 mV when illuminating the whole surface of a 3TECBT photodetector with capacitance C=1503.4 pF and size 2.792 cm x 2.792 cm. Thus, light’s intensity was P=$\frac{1}{2}$Cν${\Delta V}^{2}$=3.9 mW [1]. Since the 3TECBT photodetector’s size matched that of the *G. jamesonii* petals, the specific intensity P* on the petals was also about 3.9 mW. The radio waves had frequency ν=100 MHz. They generated a voltage of ΔV=0.355 mV when illuminating the whole surface of a 3TECBT photodetector with capacitance C=1503.4 pF and size 2.792 cm x 2.792 cm. Thus, light’s intensity was P=$\frac{1}{2}$Cν${\Delta V}^{2}$=9.5 nW [1]. Since the photodetector’s size matched that of the *G. jamesonii* petals, the specific intensity P* on the petals was also about 9.5 nW. Since the solar white light is a mixture of different frequencies, it is not possible to associate P^*^ with a unique frequency.

**Flower’ photo recording**: each selected flower was photographed with a sideview to highlight the aperture angle. We took care to photograph consistently each flower in each session. Since we were interested in the rate of petals’ closure, we focused on the angles difference for each flower between 13:30 and 16:30, rather than on the angles’ absolute value.

**Experiment years**: the results presented in this research were collected during the summers of 2023 and 2024.

**Experiment season**: the experiments were performed between mid-May and mid-August, when *G. jamesonii* were in their flowering season.

**Experiment duration**: each experiment lasted up to 10 days. The first two to three days were utilized to acclimatize the plants in their assigned environment before the selected buds would bloom. During experiment days the plants were maintained in their assigned environment.

**Experiment’ daily schedule**: in each experiment day we photographed the selected flowers at 13:30 and at 16:30 because the rate of *G. jamesonii* petals closure is rapid in this time interval.

**Experiment’ day 1**: for each selected flower, the photographing that led to useful analysis started the first day of blooming and continued for the following three days.

**Sample size of successful experiments**: 9 in solar white light, 5 in radio waves, 4 in red light, 3 in blue light, and 6 in darkness.

**Logistic limitations**: the throughput was limited by the number of available EPS enclosures and power supplies for the sources of electromagnetic waves (each light emitting diode (LED) needed one power supply while the four radio devices were powered through a power strip).

**Photography and analysis results**: The images of the photos used, and the results of the analysis are summarized in S1 Dataset.

**References**

[1] Rybarczyk RJ, Federick AED, Kokhan O, Luckay R, Scarel G. Probing electromagnetic wave energy with an in-series assembly of thermoelectric devices. AIP Adv. 2022 Apr 1;12(4):045201.
