# Supplementary material for: A phenomenological comparison of the effects of blue light, red light and radio waves on the escape speed of Caenorhabditis elegans and the rate of closure of Gerbera jamesonii petals
Source: PLoS One. 2026 Apr 1;21(4):e0343498. doi: 10.1371/journal.pone.0343498 (PMC13043045; doi:10.1371/journal.pone.0343498)

This file contains tables and images with the data that enabled the creation of Figure 6 in the Main Text of "*A phenomenological comparison of the effects of blue light, red light and radio waves on the escape speed of Caenorhabditis elegans and the rate of closure of Gerbera jamesonii petals*", Kline *et al*.

Plants from which selected *G. jamesonii* were selected

Darkness	Blue light	Red light	Radio waves	Solar white light
33-A-d	40-B-b	35-A-r	32-A-4xr	31-A-w
36-A-d	46-A-b	35-B-r	41-B-4xr	34-A-w
41-A-d	53-A-b	48-C-r	49-A-4xr	34-B-w
47-B-d		55-A-r	49-B-4xr	37-A-w
50-A-d			52-A-4xr	37-B-w
50-B-d				48-A-w
				50-C-w
				50-D-w
				51-A-w

50 → order in which the plant was purchased

B → label of the flower chosen in a specific plant

d → environment of plant during the experiment

Angles and rate of closures on Day 1

Darkness	Blue light	Red light	Radio waves	Solar white light
138.5°/111.5° 0.15°/min	129°/116.5° 0.069°/min	86.5°/91.5° -0.028°/min	160.5°/99.5° 0.34°/min	170°/132° 0.21°/min
113°/113.5° -0.0028°/min	144.5°/136° 0.047°/min	94°/98° -0.022°/min	200°/151° 0.27°/min	107°/72.5° 0.19°/min
145°/131° 0.078°/min	117°/103.5° 0.074°/min	87°/92° -0.028°/min	140°/107.5° 0.18°/min	139°/104° 0.195°/min
102°/94.5° 0.042°/min		80°/87° -0.039°/min	113°/97° 0.089°/min	155°/86° 0.38°/min
144°/135.5° 0.047°/min			79°/47° 0.18°/min	219°/139° 0.45°/min
130°/116° 0.078°/min				110°/87° 0.128°/min
				148°/77.5° 0.392°/min
				159.5°/70° 0.497°/min
				136°/76.5° 0.33°/min
**0.065±0.051**	**0.063±0.014**	**-0.029±0.007**	**0.212±0.096**	**0.308±0.131**

angle at 13:30

angle at 16:30

rate of closure

Average rate of closure (°/min)

Angles and rate of closures on Day 2

	Darkness	Blue light	Red light	Radio waves	Solar white light
	139°/132.5° 0.036°/min	151°/117° 0.18°/min	92°/104° -0.067°/min	154°/155° -0.005°/min	166°/138° 0.15°/min
	124°/119° 0.0028°/min	164°/155.5° 0.047°/min	120°/120° 0°/min	195°/169.5° 0.14°/min	133°/106° 0.15°/min
	170°/147° 0.127°/min	144°/137° 0.039°/min	97.5°/104° -0.036°/min	155.5°/143.5° 0.067°/min	139.5°/112° 0.153°/min
	132.5°/142° -0.042°/min		121°/141° -0.11°/min	158°/130° 0.155°/min	173°/148° 0.14°/min
	167°/165.5° 0.0083°/min			131.5°/81° 0.28°/min	206°/156° 0.28°/min
	144.5°/141.5° 0.017°/min				140°/124° 0.089°/min
					173°/125° 0.27°/min
					165°/124.5° 0.225°/min
					139°/78° 0.34°/min
**Average rate of closure (°/min)**	**0.025±0.056**	**0.087±0.079**	**-0.053±0.047**	**0.127±0.106**	**0.200±0.083**

Angles and rate of closures on Day 3

Darkness	Blue light	Red light	Radio waves	Solar white light
111.5°/110° 0.0083°/min	151.5°/135° 0.092°/min	101.5°/110° -0.05°/min	141°/150° -0.05°/min	148°/119.5° 0.158°/min
118°/112° 0.033°/min	167°/160.5° 0.036°/min	126°/110° 0.09°/min	175°/158° 0.097°/min	133°/110° 0.13°/min
170°/141.5° 0.16°/min	-	107°/111° -0.0194°/min	139°/166.5° -0.152°/min	131°/110° 0.12°/min
134.5°/142.5° -0.044°/min		-	169°/180° -0.061°/min	162.5°/141° 0.12°/min
138.5°/150° -0.064°/min			-	187°/144° 0.24°/min
191°/188.5° 0.014°/min				143°/139.5° 0.0194°/min
				172°/137° 0.194°/min
				164°/119° 0.25°/min
				140°/88° 0.29°/min
**0.018±0.079**	**0.064±0.039**	**-0.007±0.074**	**-0.041±0.103**	**0.169±0.083**

Average rate of closure (°/min): the bottom row of the table.

Angles and rate of closures on Day 4

Darkness	Blue light	Red light	Radio waves	Solar white light
112°/106°<br>0.033°/min	155.5°/143°<br>0.069°/min	99.5°/91°<br>0.014°/min	126°/140°<br>-0.078°/min	147°/129°<br>0.1°/min
122.5°/116.5°<br>0.039°/min	157.5°/155.5°<br>0.011°/min	113.5°/108°<br>0.03°/min	165°/149°<br>0.089°/min	137°/116°<br>0.12°/min
144°/134°<br>0.05°/min	-	123°/121.5°<br>0.0083°/min	136.5°/154.5°<br>-0.1°/min	139°/119°<br>0.11°/min
128°/131°<br>-0.017°/min		-	131°/150°<br>-0.105°/min	151.5°/126°<br>0.14°/min
149.5°/156°<br>-0.036°/min			-	181.5°/160°<br>0.12°/min
190°/190.5°<br>-0.0003°/min				150°/118°<br>0.178°/min
				183°/131°<br>0.29°/min
				143°/108°<br>0.194°/min
				-
**0.011±0.034**	**0.040±0.041**	**0.017±0.011**	**-0.048±0.092**	**0.156±0.059**

Average rate of closure (°/min)

G. jamesonii in dark

Dates: 6/30/23 – 7/3/23, Flower: 33-A-d

13:30

16:30

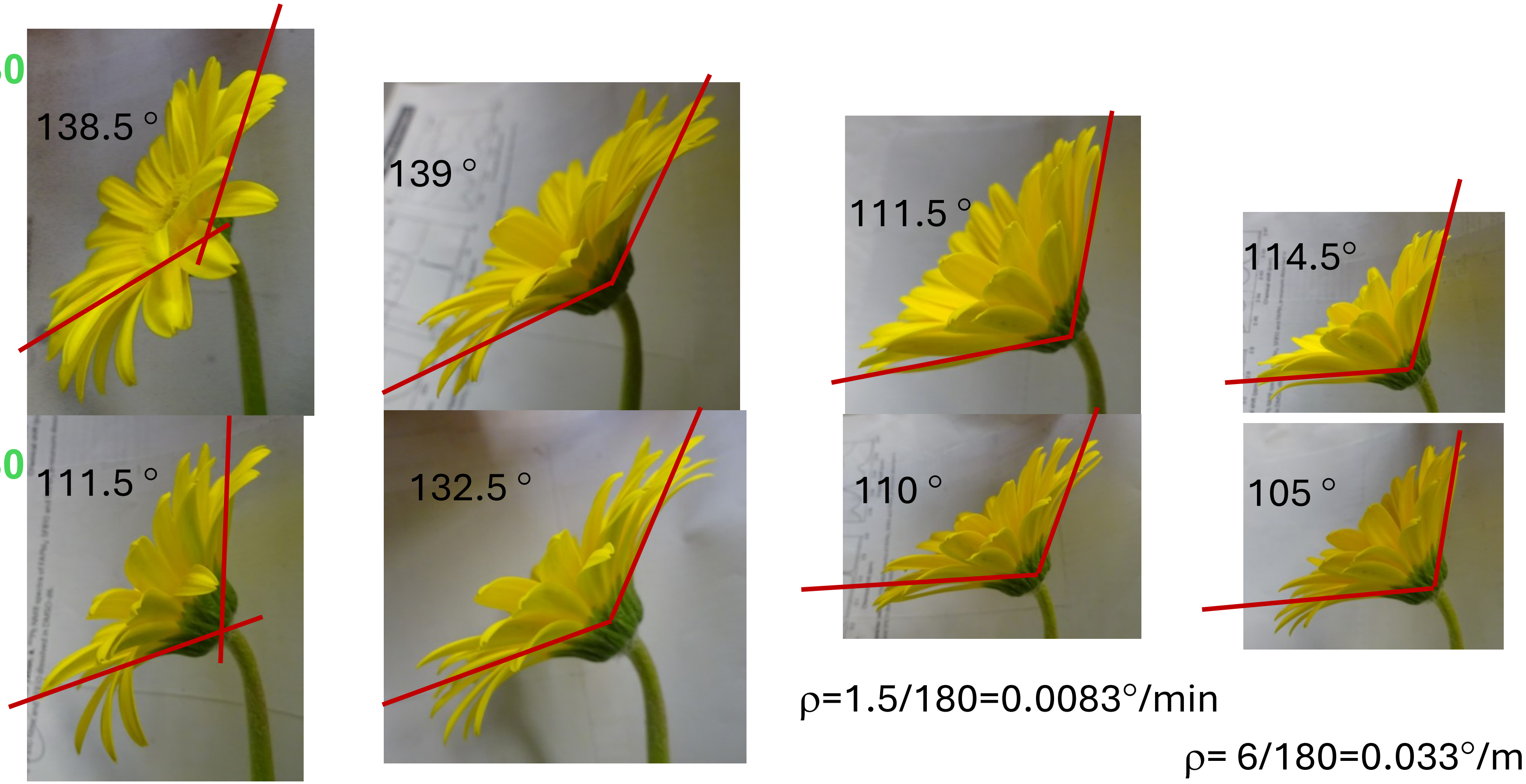

ρ=1.5/180=0.0083°/min

ρ= 6/180=0.033°/min

ρ=27/180=0.15 °/min

ρ=6.5/180=0.036 °/min

**Day 1**	**Day 2**	**Day 3**	**Day 4**	**Day of blossom**

Time of Day

Dates: 7/9-12/23, Flower: 36-A-d

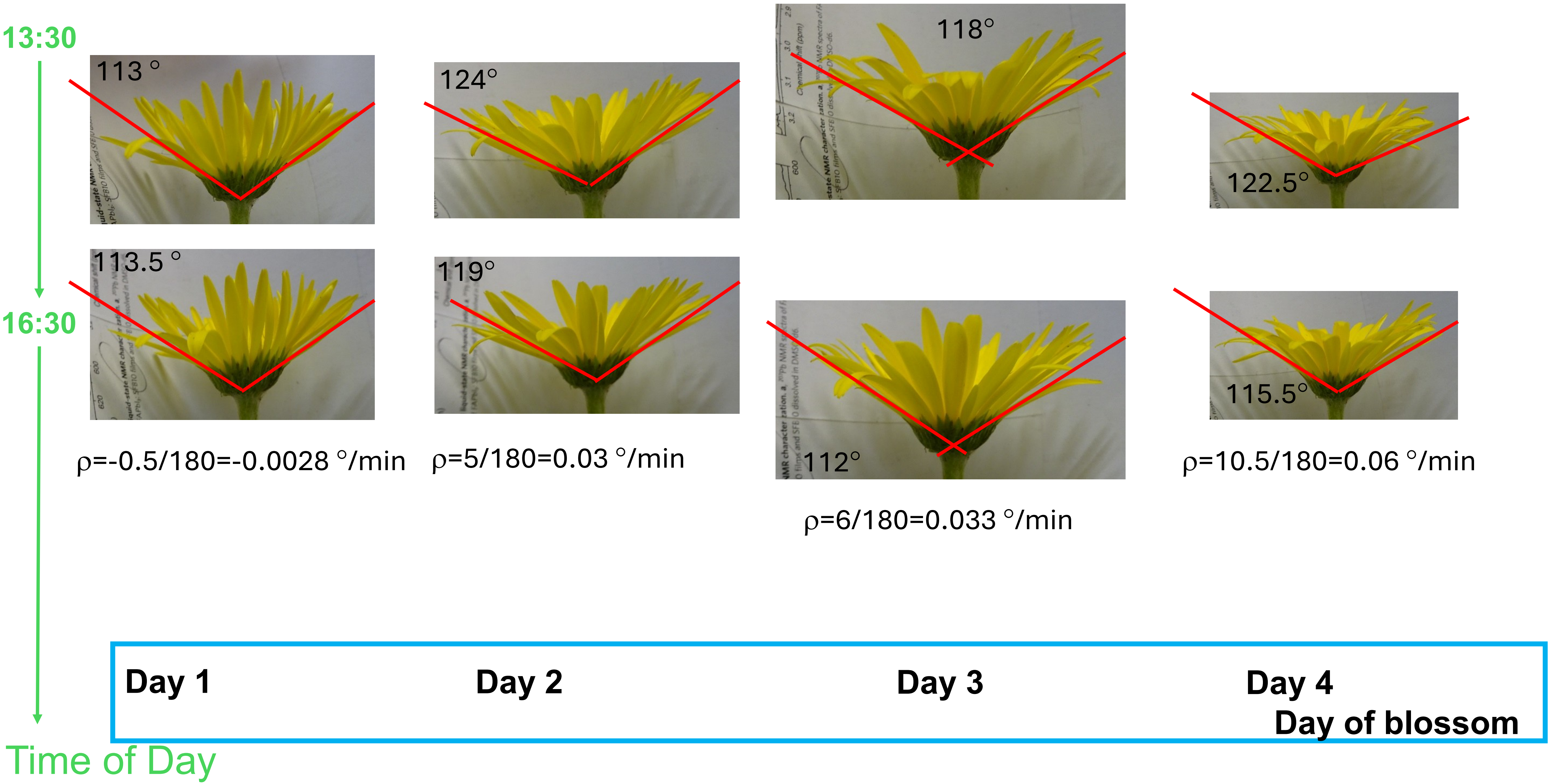

Dates: 7/30/23 – 8/2/23, Flower: 41-A-d

13:30

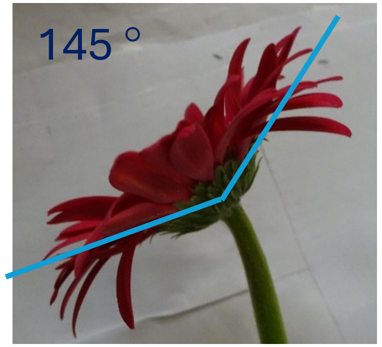

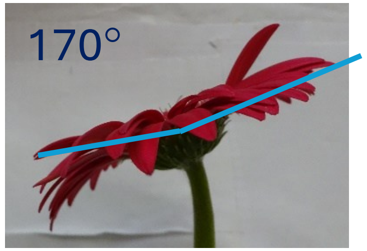

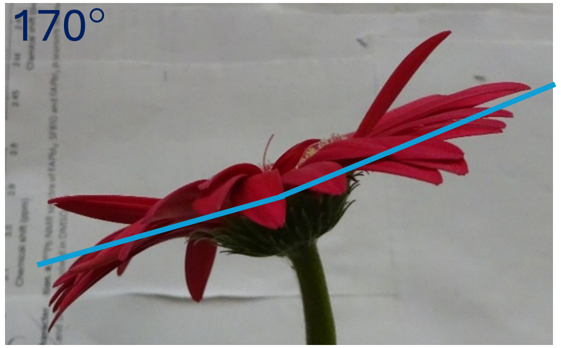

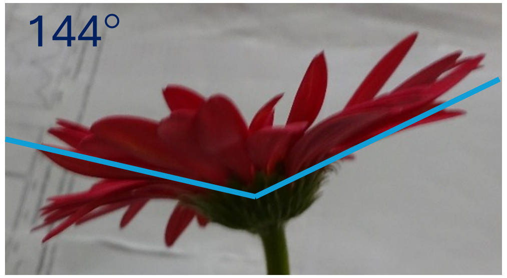

16:30

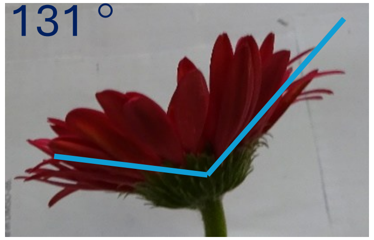

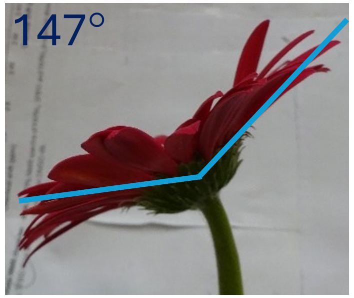

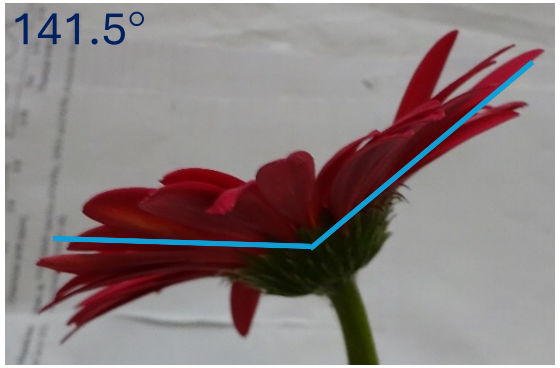

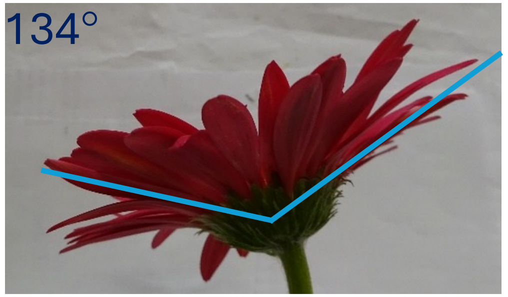

$\rho=14/180=0.078$ °/min

$\rho=23/180=0.127$ °/min

$\rho=28.5/180=0.16$ °/min

$\rho=10/180=0.05$ °/min

**Day 1**	**Day 2**	**Day 3**	**Day 4**	**Day of blossom**

Time of Day

Dates: 6/15-18/24, Flower: 47-B-d

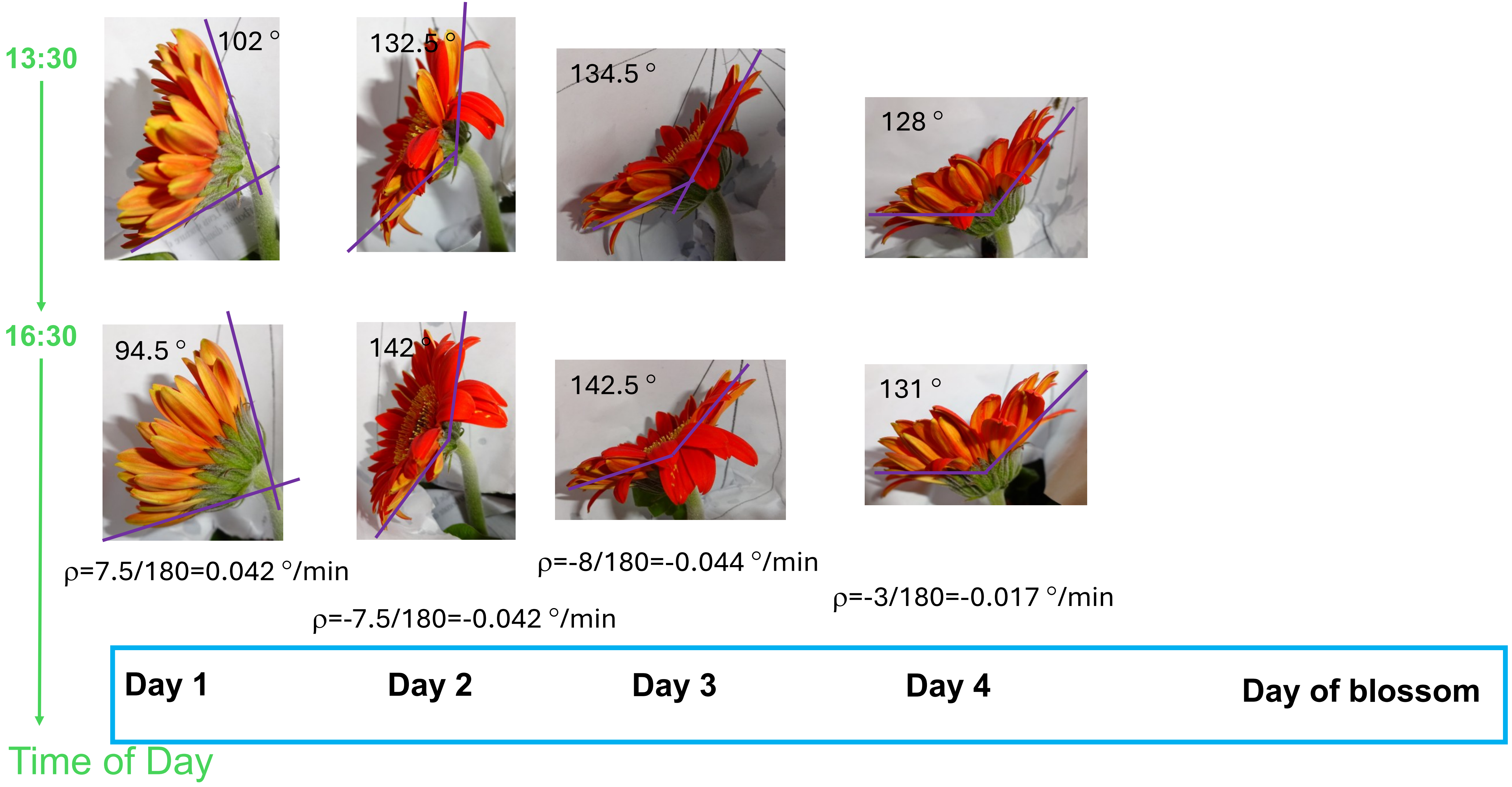

Dates: 6/29/24 – 7/2/24, Flower: 50-A-d

13:30

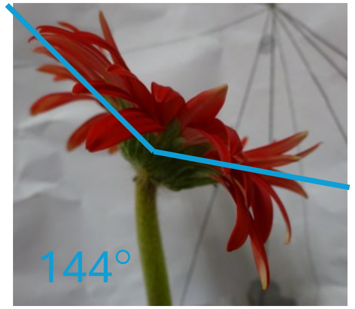

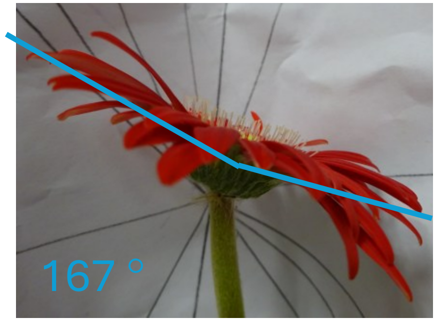

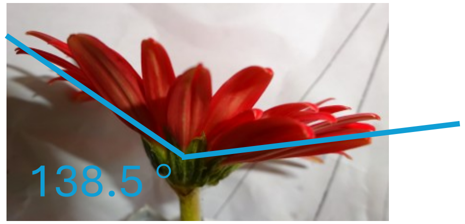

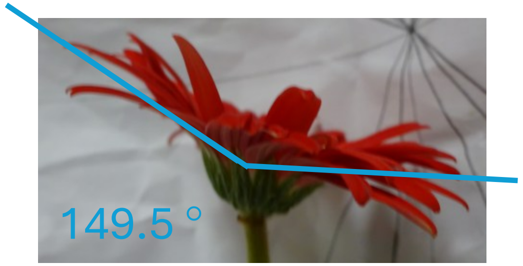

16:30

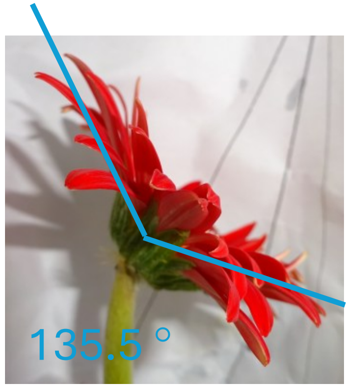

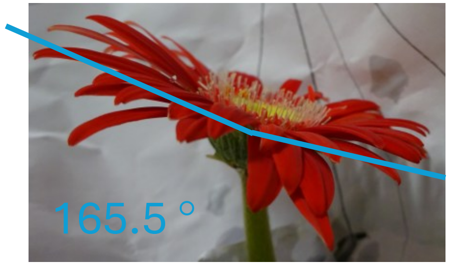

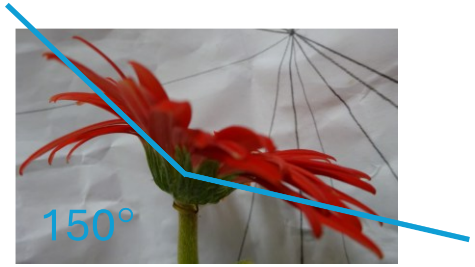

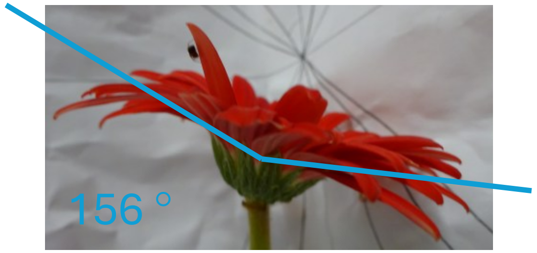

ρ=8.5/180=0.047 °/min

ρ=1.5/180=0.0083 °/min

ρ=-11.5/180=-0.064 °/min

ρ=-6.5/180=-0.036 °/min

Day 1	Day 2	Day 3	Day 4	Day of blossom

Time of Day

Dates: 6/30/24 – 7/3/24, Flower: 50-B-d

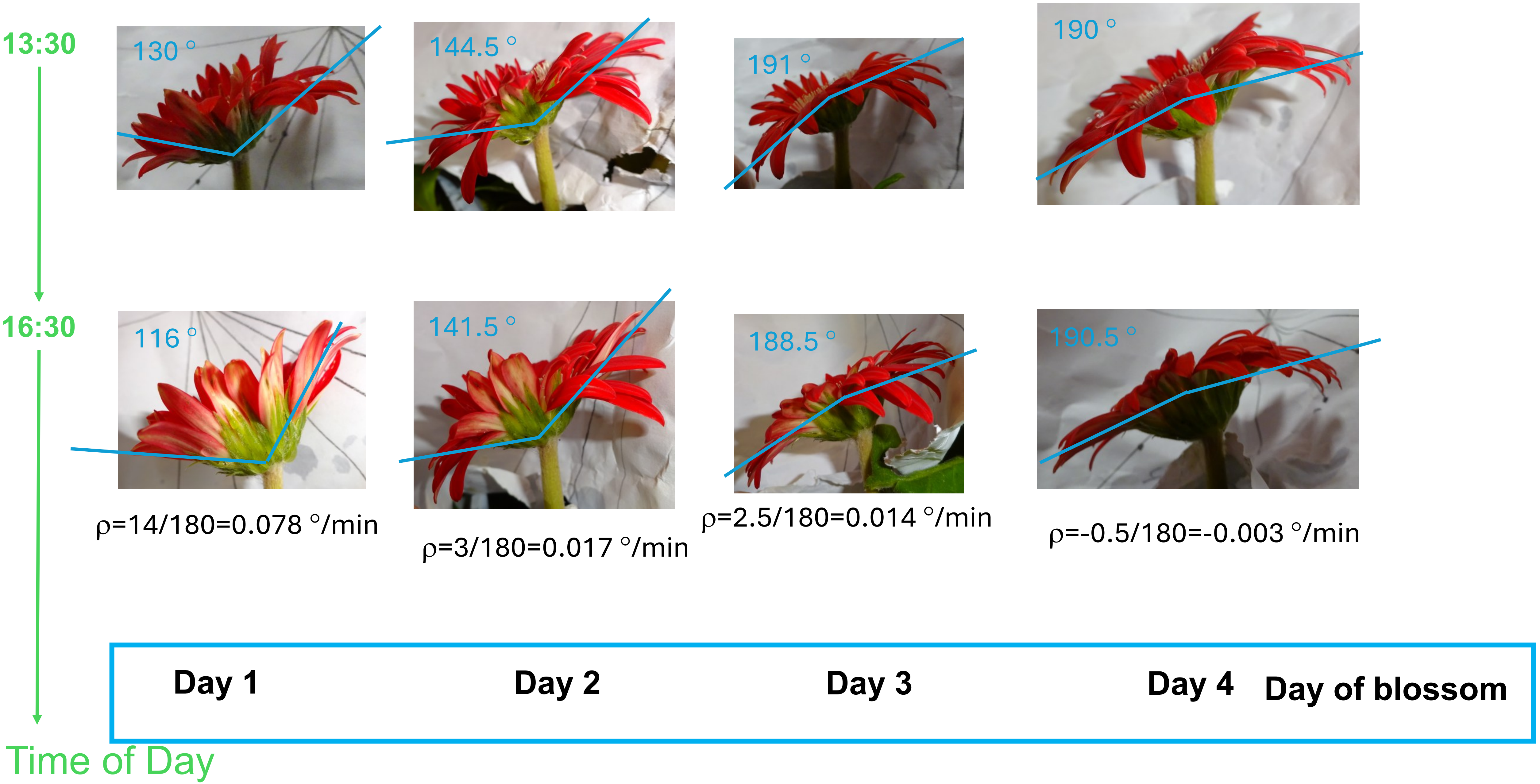

G. jamesonii in blue light

Dates: 7/30/24 – 8/2/24, Flower: 40-B-b

13:30

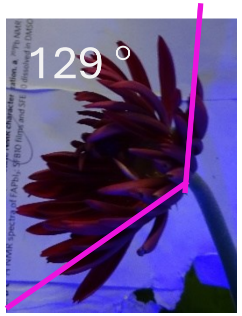

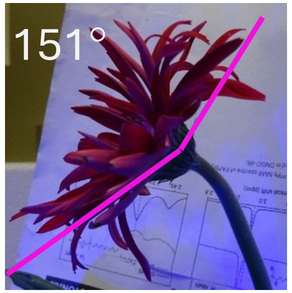

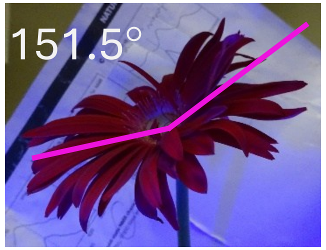

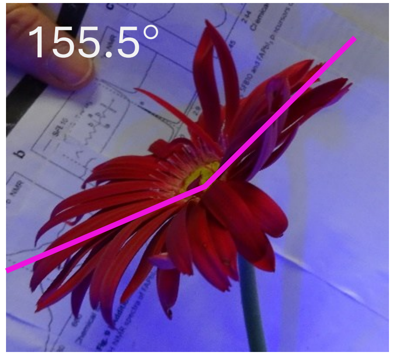

16:30

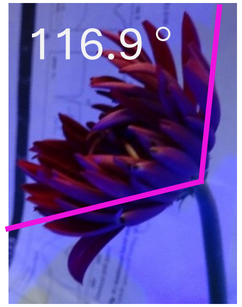

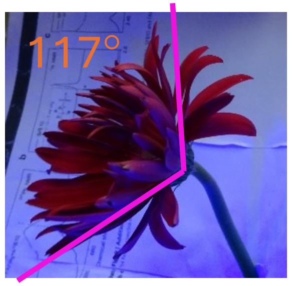

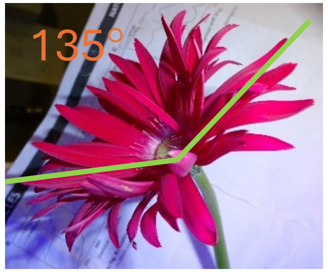

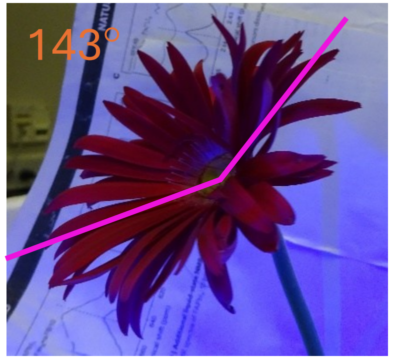

ρ=12.5/180=0.069 °/min

ρ=34/180=0.18 °/min

ρ=16.5/180=0.092 °/min

ρ=12.5/180=0.069 °/min

**Day 1**	**Day 2**	**Day 3**	**Day 4**	**Day of blossom**

Time of Day

Dates: 6/16-19/24, Flower: 46-A-b

13:30

16:30

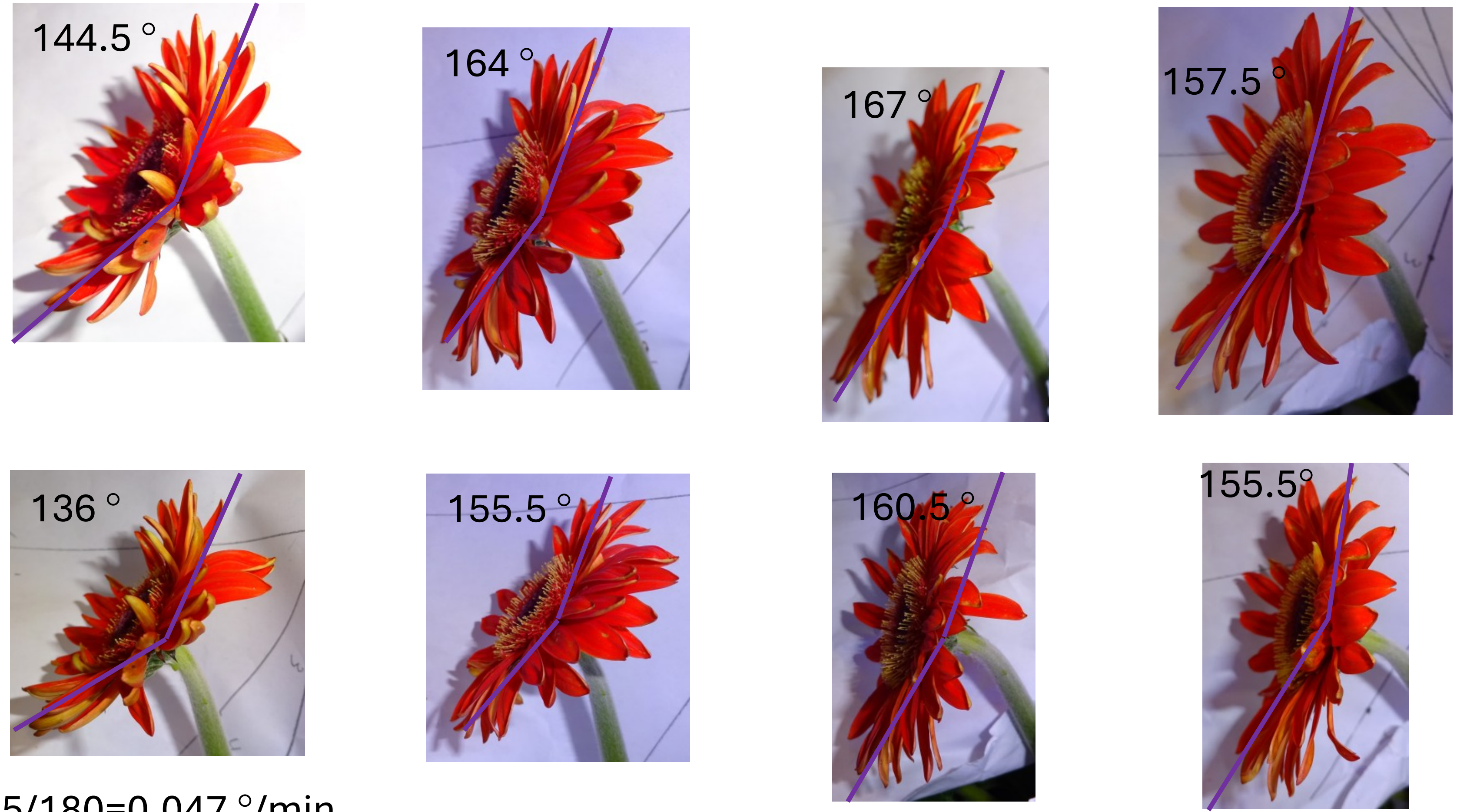

ρ=8.5/180=0.047 °/min

ρ=8.5/180=0.047 °/min

ρ=6.5/180= 0.036 °/min

ρ=2/180=0.011 °/min

Day 1	Day 2	Day 3	Day 4	Day of blossom

Time of Day

Dates: 7/16-17/24, Flower: 53-A-b

13:30

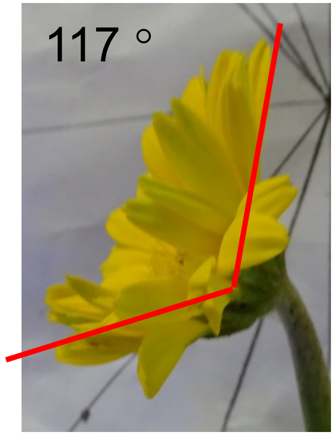

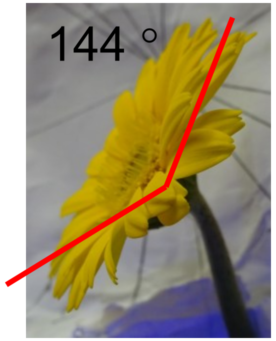

16:30

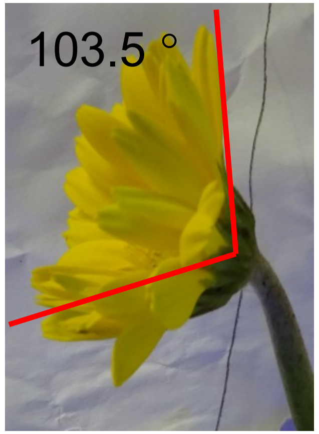

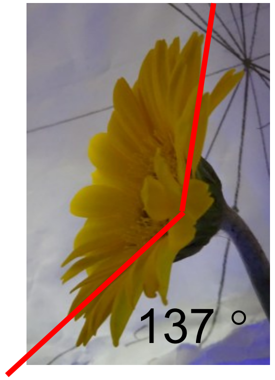

ρ=7/180=0.039 °/min

ρ=13.5/180=0.075 °/min

Day 1 **Day 2** **Day of blossom**

Time of Day

G. jamesonii in red light

Dates: 7/8-11/23, Flower: 35-A-r

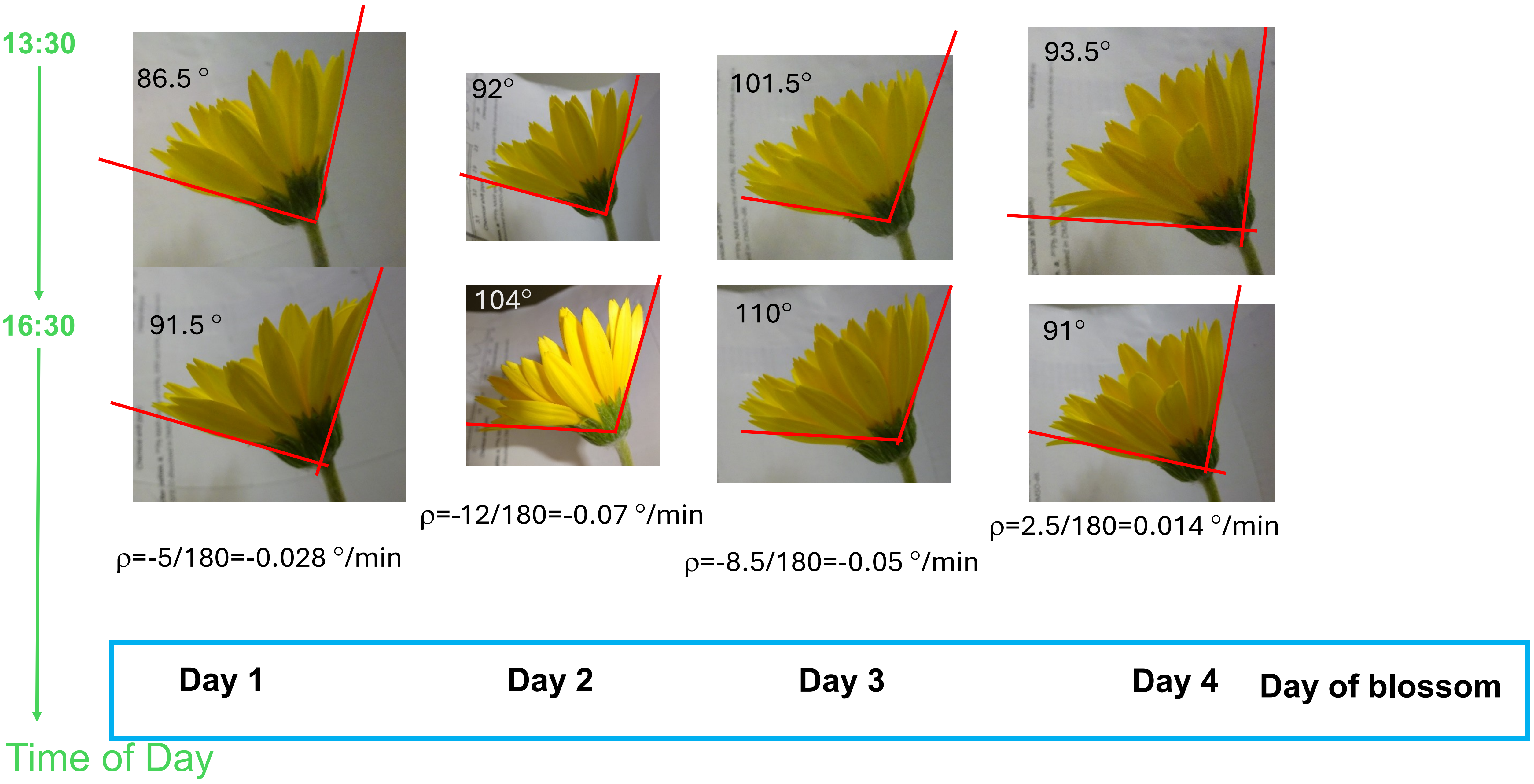

Dates: 7/9-12/23, Flower: 35-B-r

13:30

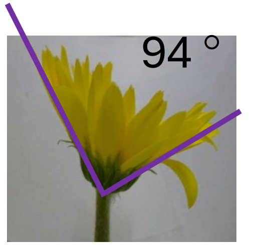

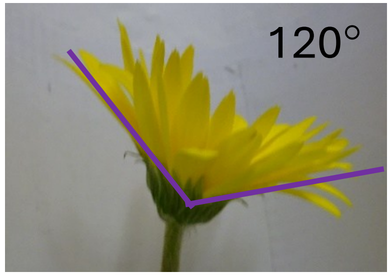

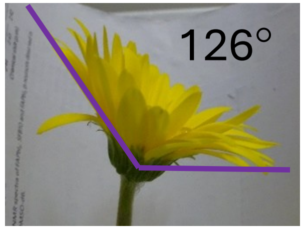

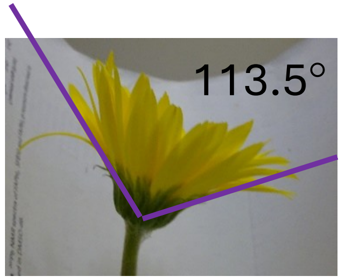

16:30

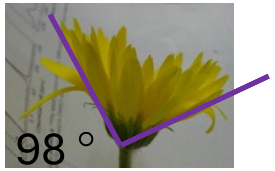

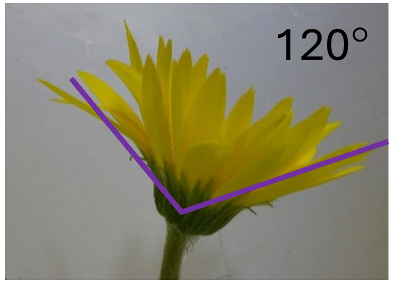

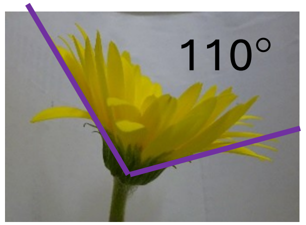

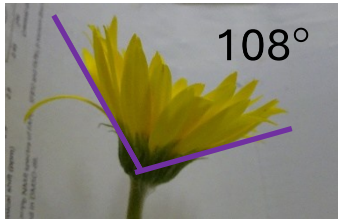

ρ=-4/180=-0.022 °/min

ρ=0/180=0 °/min

ρ=16/180=0.09 °/min

ρ=5.5/180=0.03 °/min

**Day 1**	**Day 2**	**Day 3**	**Day 4**	**Day of blossom**

Time of Day

Dates: 7/8-11/24, Flower: 48-C-r

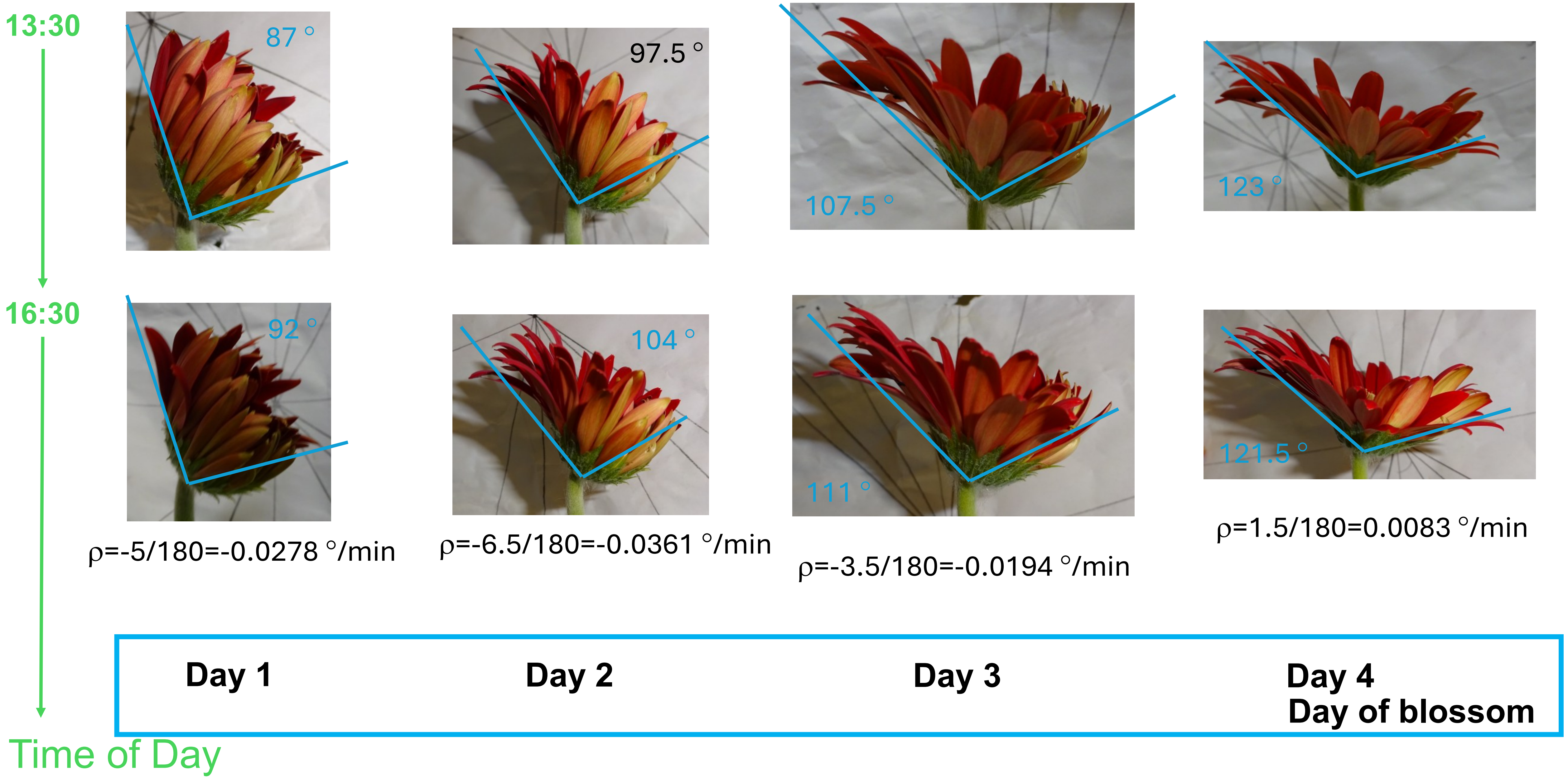

Dates: 7/16-17/24, Flower: 55-A-r

13:30

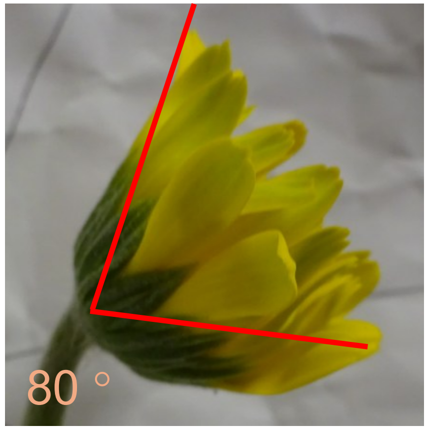

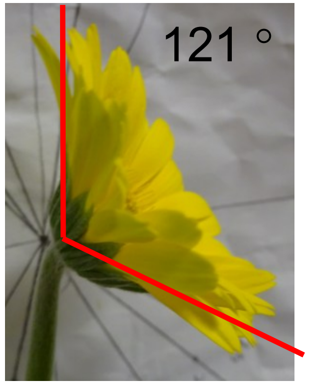

16:30

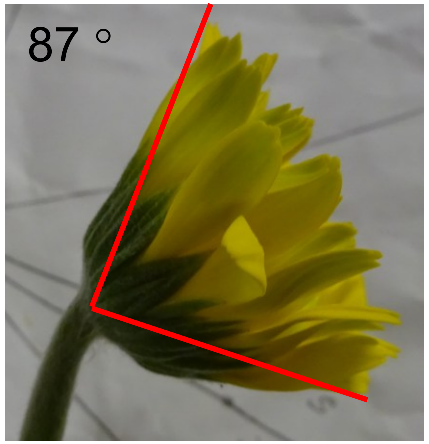

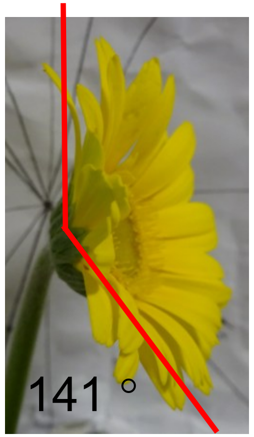

ρ=-7/180=-0.039 °/min

ρ=-20/180=-0.11 °/min

Day 1 **Day 2** **Day of blossom**

Time of Day

G. jamesonii in radio waves

Dates: 6/30/23 – 7/3/23, Flower: 32-A-4xr

13:30

16:30

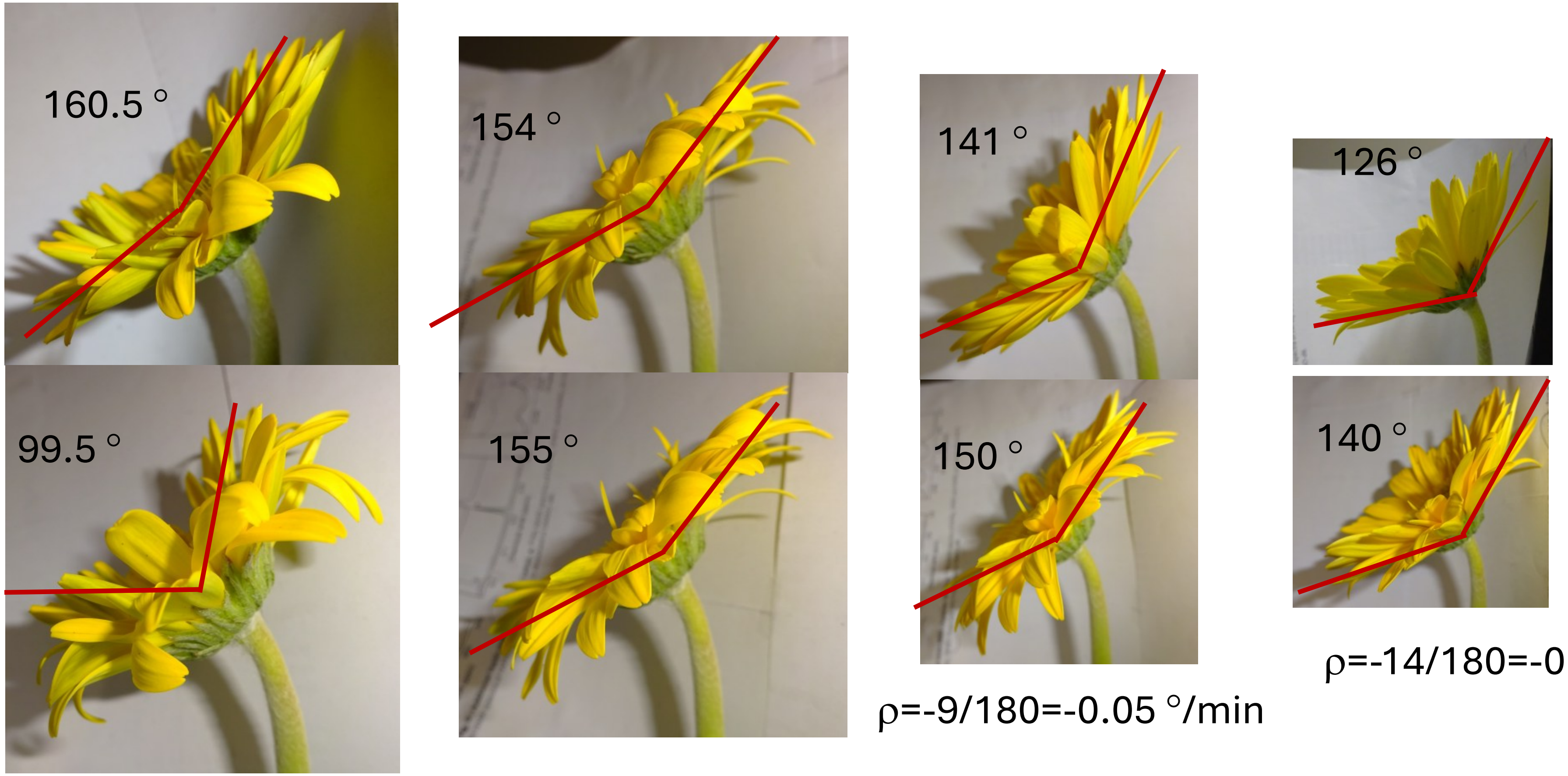

ρ=61/180=0.34 °/min

ρ=-1/180=-0.0055 °/min

ρ=-9/180=-0.05 °/min

ρ=-14/180=-0.078 °/min

Day 1	Day 2	Day 3	Day 4	Day of blossom

Time of Day

Dates: 8/14-17/23, Flower: 41-B-4xr

13:30

16:30

16:30

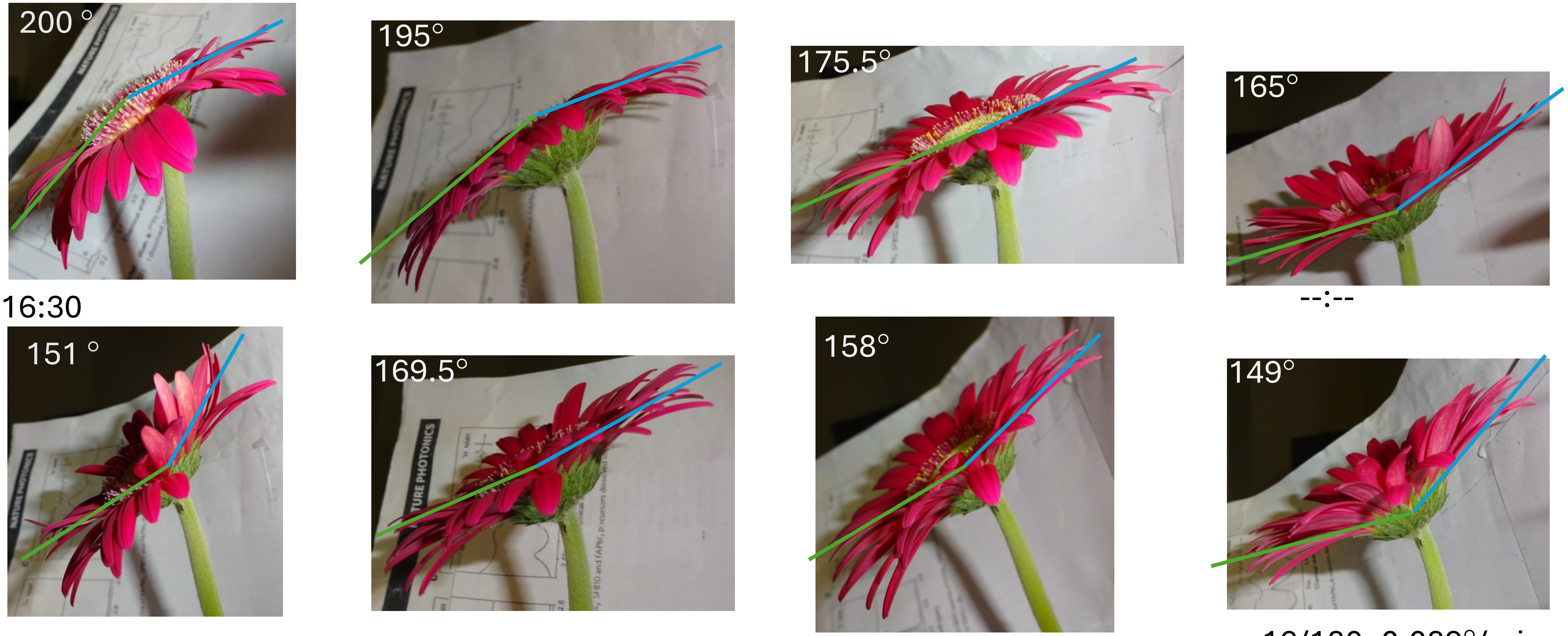

--:--

ρ=49/240=0.27 °/min

ρ=25.5/180=0.14°/min

ρ=17.5/180=0.097°/min

ρ=16/180=0.089°/min

Day 1 **Day 2** **Day 3** **Day 4**

Day of blossom

Time of Day

Dates: 6/29/24 – 7/2/24, Flower: 49-A-4xr

13:30

16:30

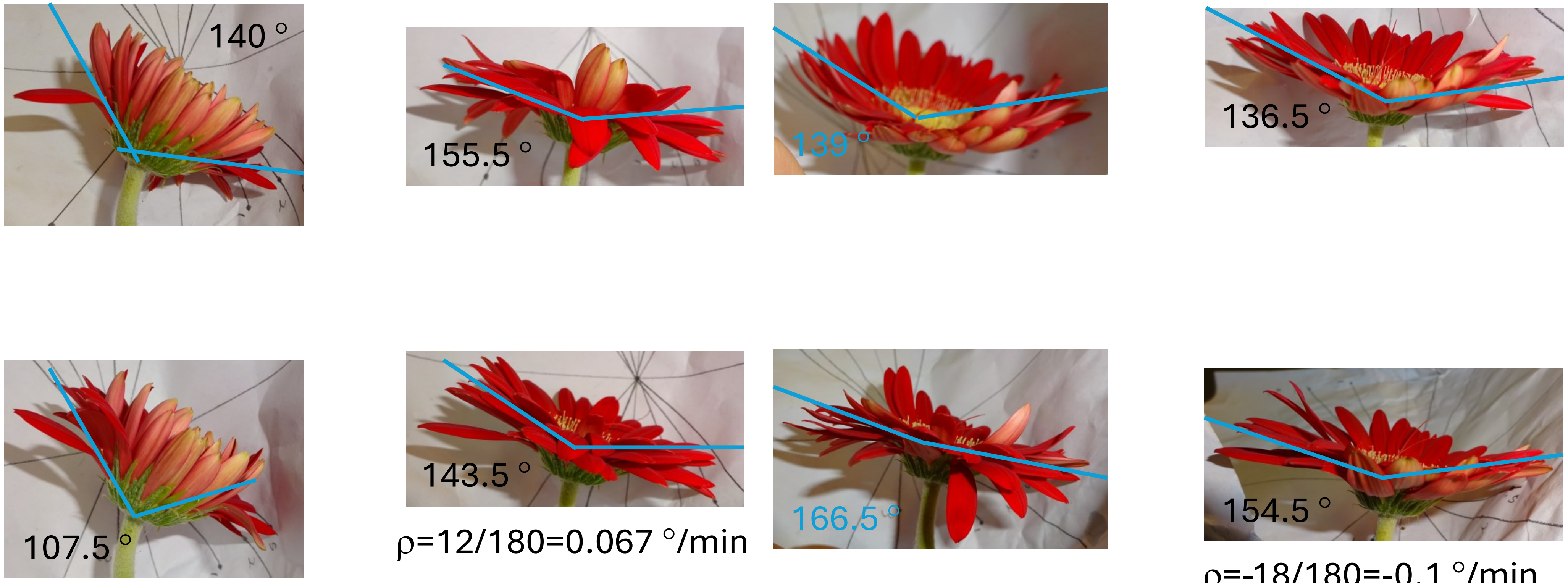

ρ=32.5/180=0.18 °/min

ρ=12/180=0.067 °/min

ρ=-27.5/180=-0.152 °/min

ρ=-18/180=-0.1 °/min

Day 1	Day 2	Day 3	Day 4	Day of blossom

Time of Day

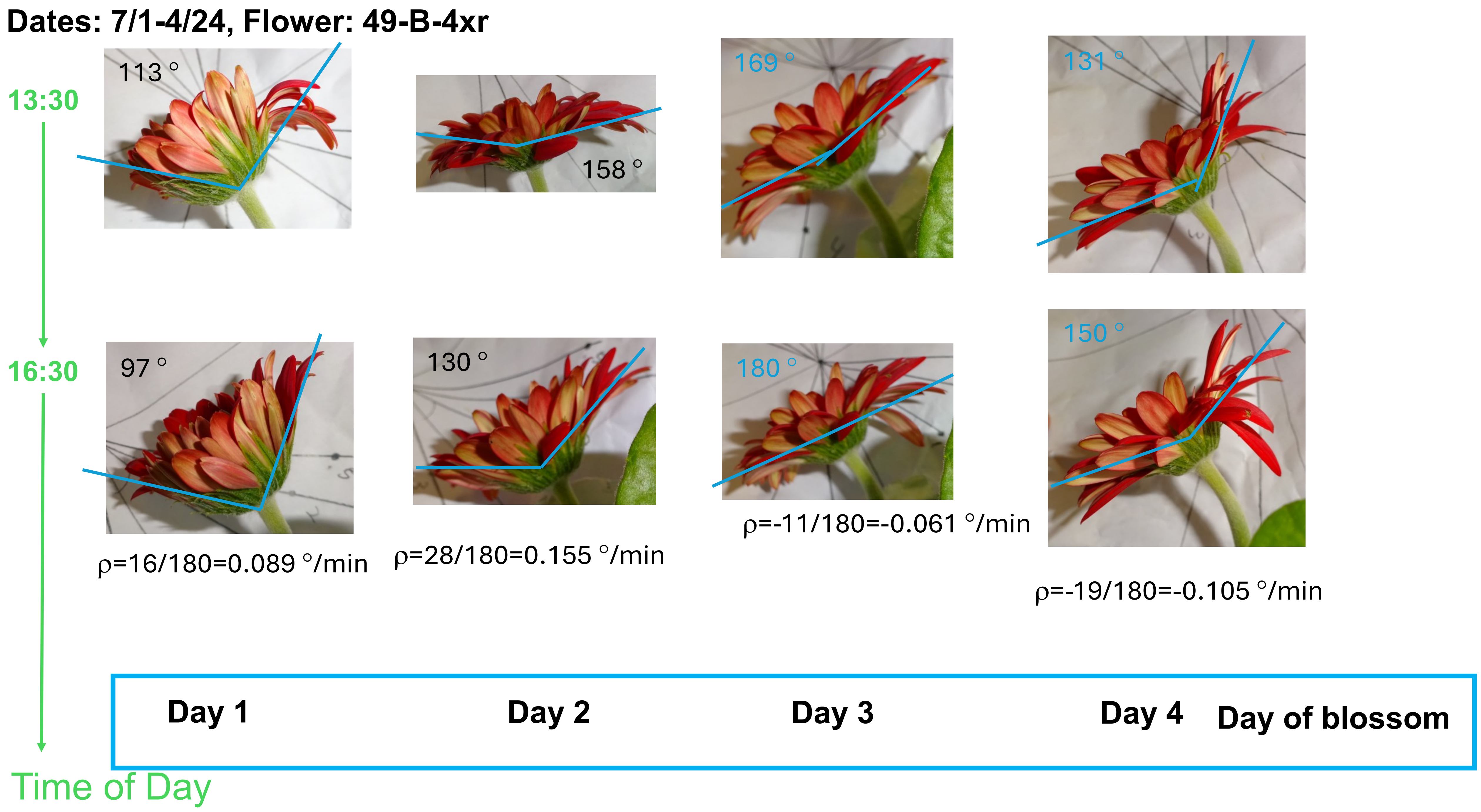
Dates: 7/1-4/24, Flower: 49-B-4xr
13:30
16:30
Time of Day
113 °
158 °
169 °
131 °
97 °
130 °
180 °
150 °
ρ=16/180=0.089 °/min
ρ=28/180=0.155 °/min
ρ=-11/180=-0.061 °/min
ρ=-19/180=-0.105 °/min
Day 1
Day 2
Day 3
Day 4
Day of blossom

Dates: 7/16-17/24, Flower: 52-A-4xr

13:30

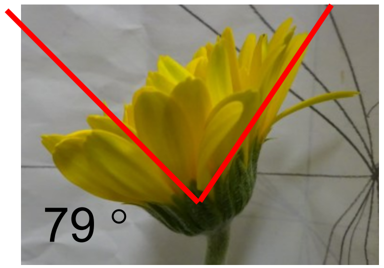

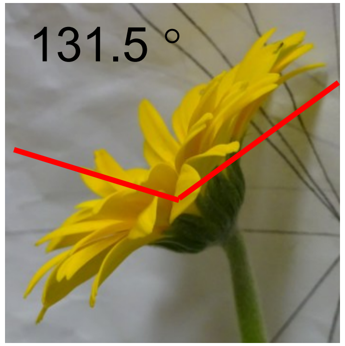

16:30

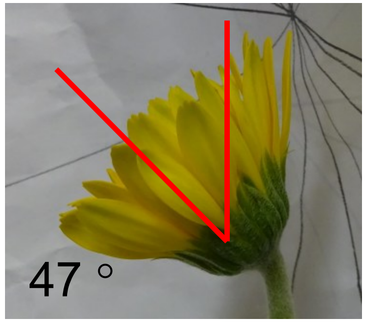

ρ=32/180=0.18 °/min

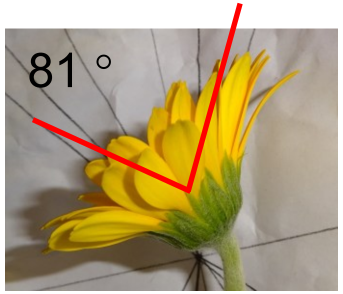

ρ=50.5/180=0.28 °/min

**Day 1**	**Day 2**	**Day of blossom**

Time of Day

G. jamesonii in solar white light

Dates: 6/30/23 – 7/3/23, Flower: 31-A-w

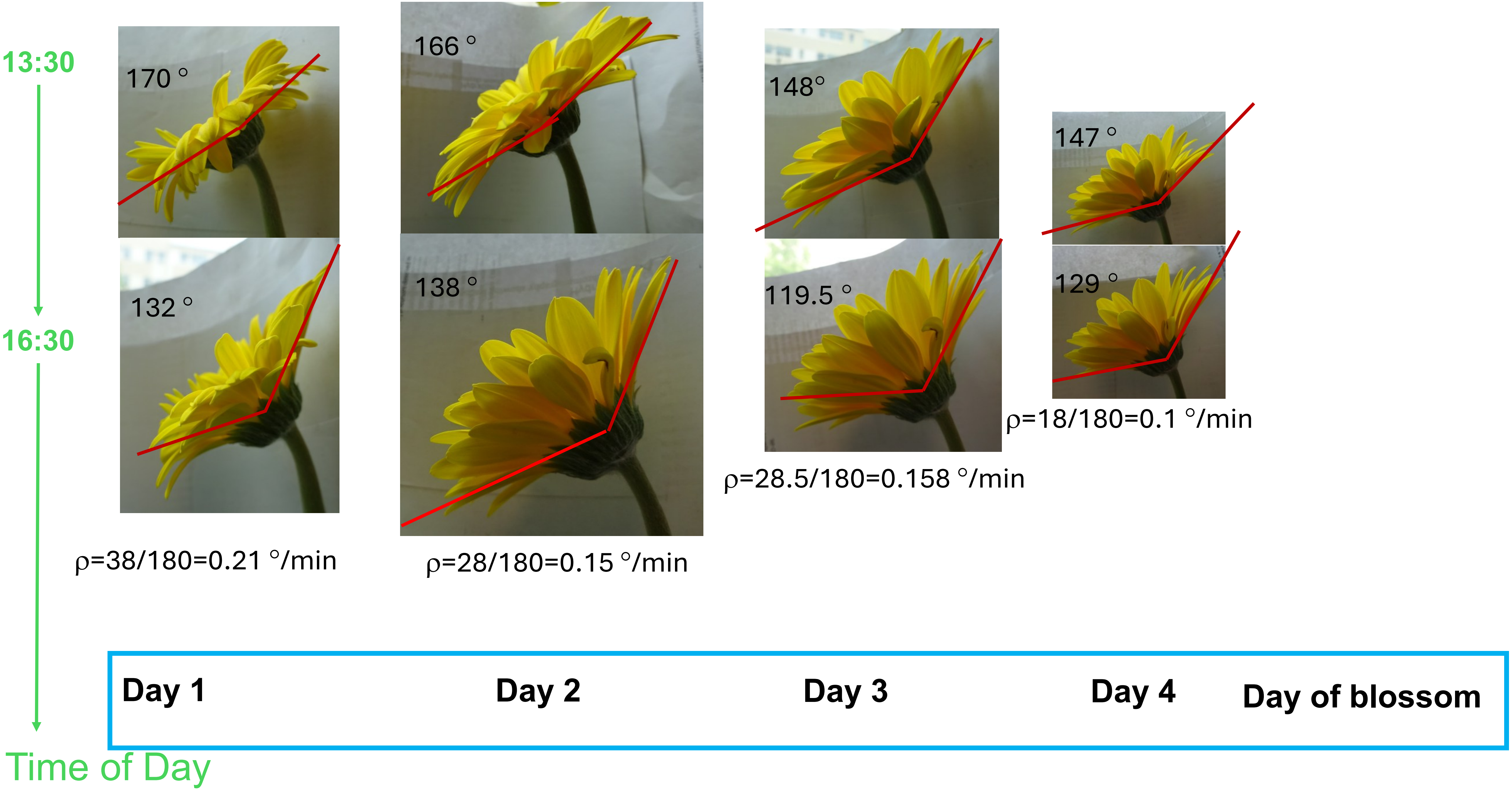

Dates: 7/8-11/23, Flower: 34-A-w

13:30

16:30

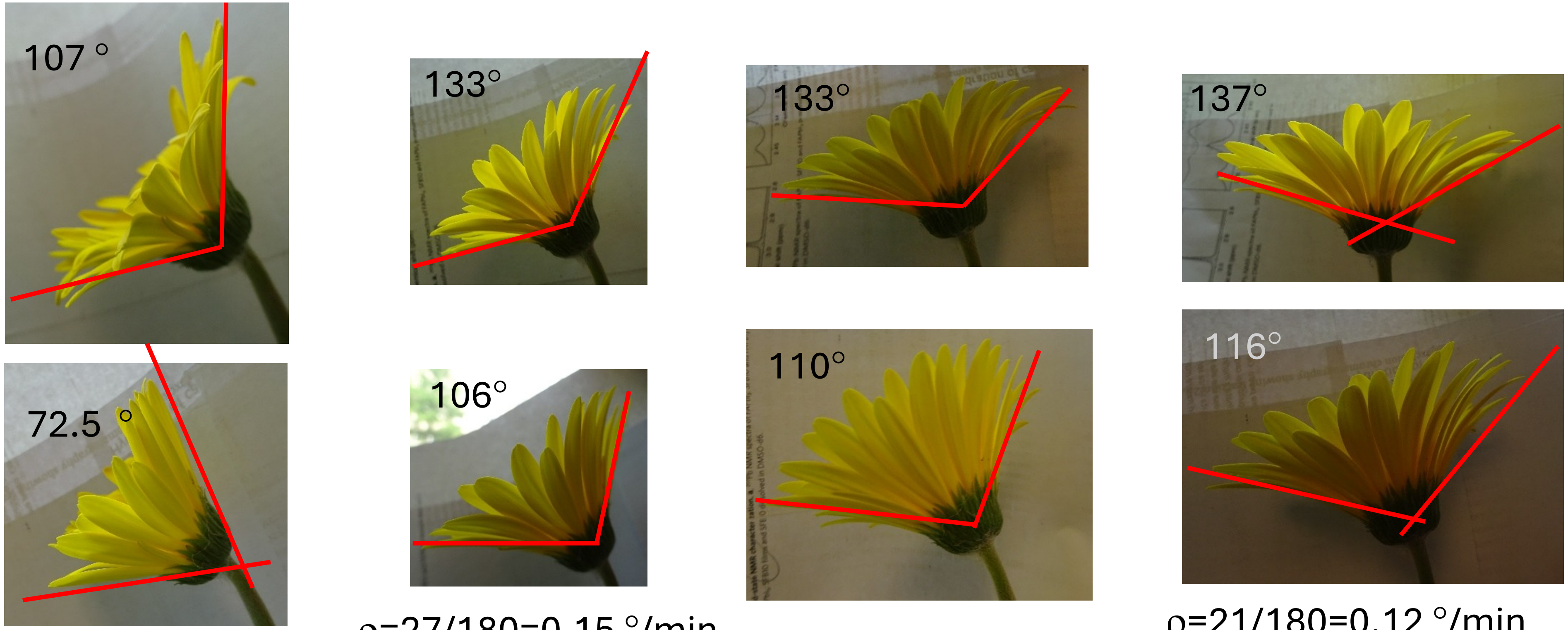

ρ=34.5/180=0.19 °/min

ρ=27/180=0.15 °/min

ρ=23/180=0.13 °/min

ρ=21/180=0.12 °/min

Day 1	Day 2	Day 3	Day 4	Day of blossom

Time of Day

Dates: 7/9-12/23, Flower: 34-B-w

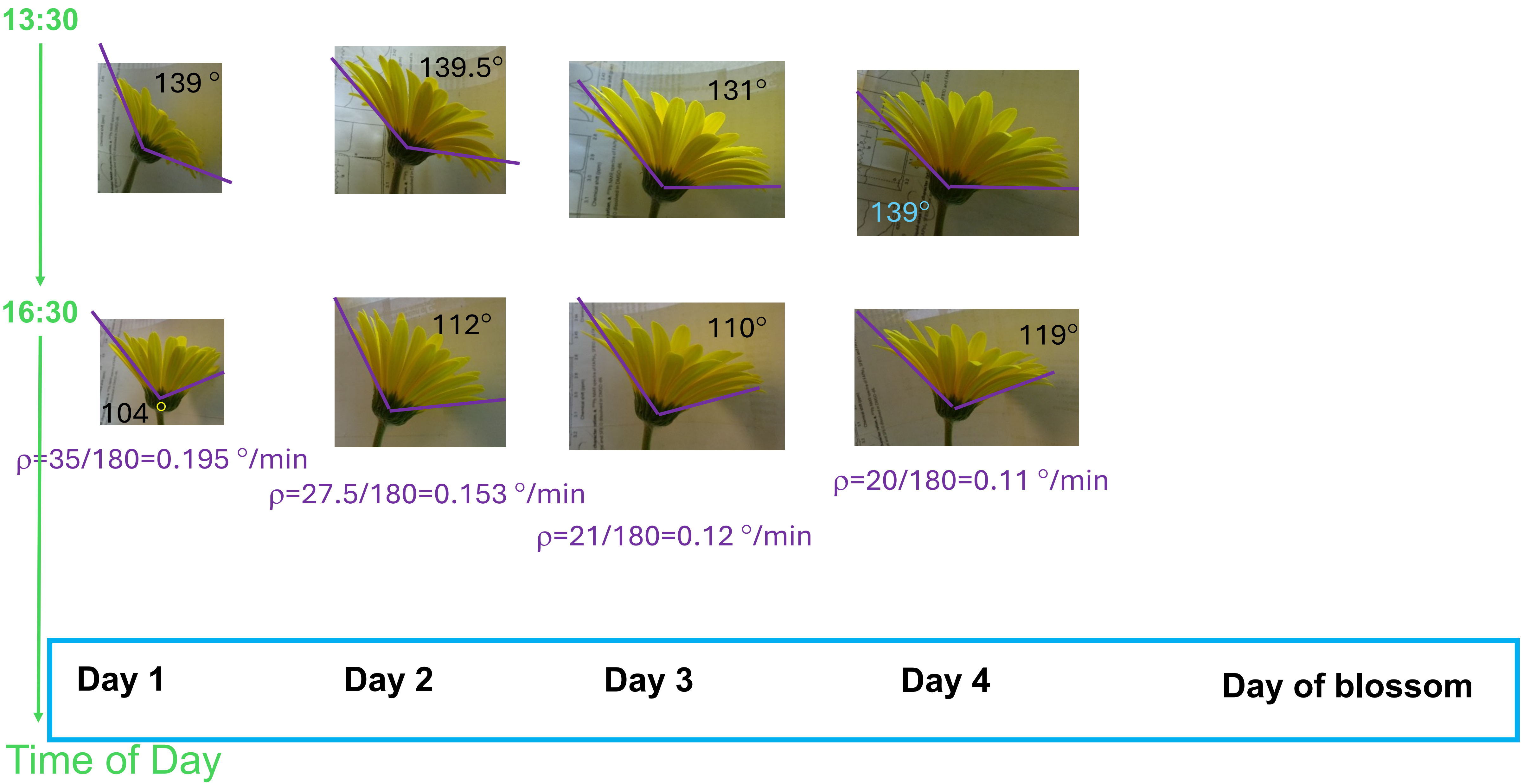

Dates: 7/15-18/23, Flower: 37-A-w

13:30

16:30

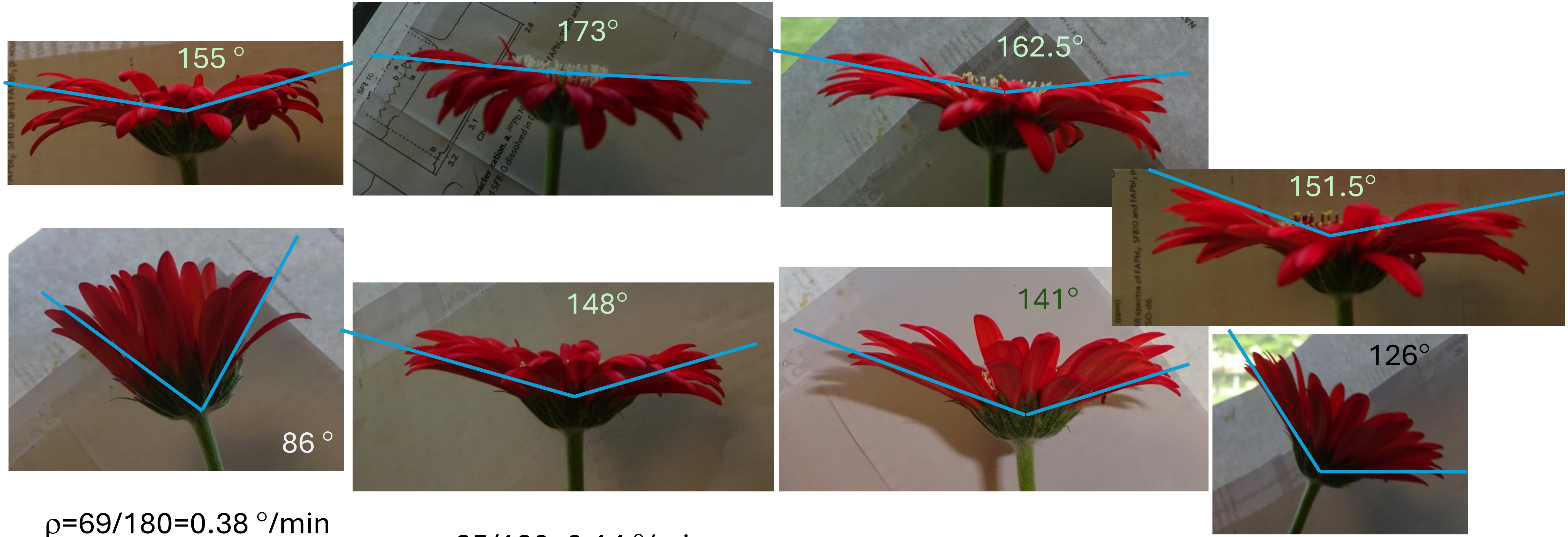

ρ=69/180=0.38 °/min

ρ=25/180=0.14 °/min

ρ=21.5/180=0.12 °/min

ρ=25.5/180=0.14 °/min

Day 1 **Day 2** **Day 3** **Day 4**

Day of blossom

Time of Day

Dates: 7/16-19/23, Flower: 37-B-w

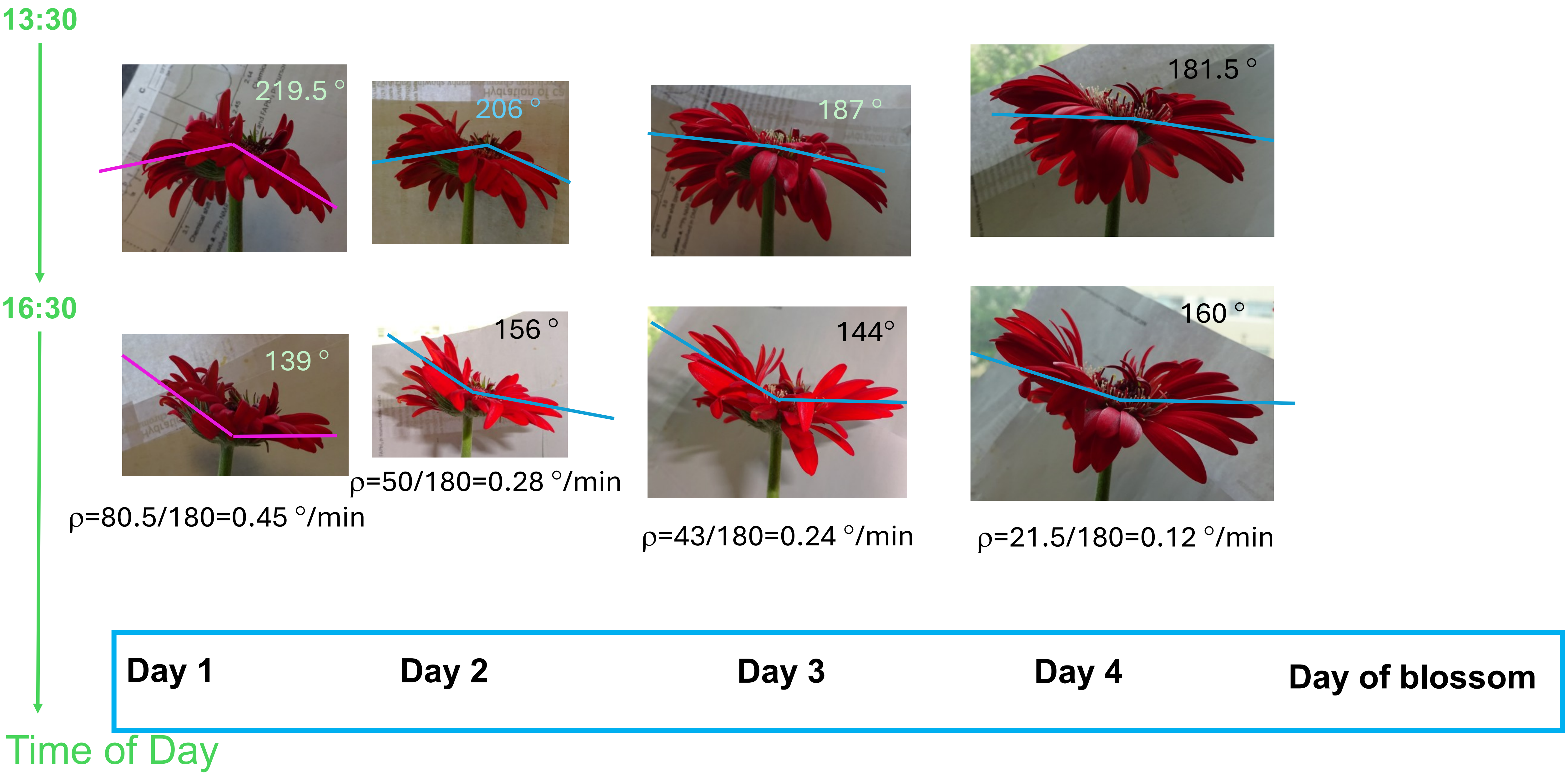

Dates: 6/28/23 – 7/1/23, Flower: 48-A-w

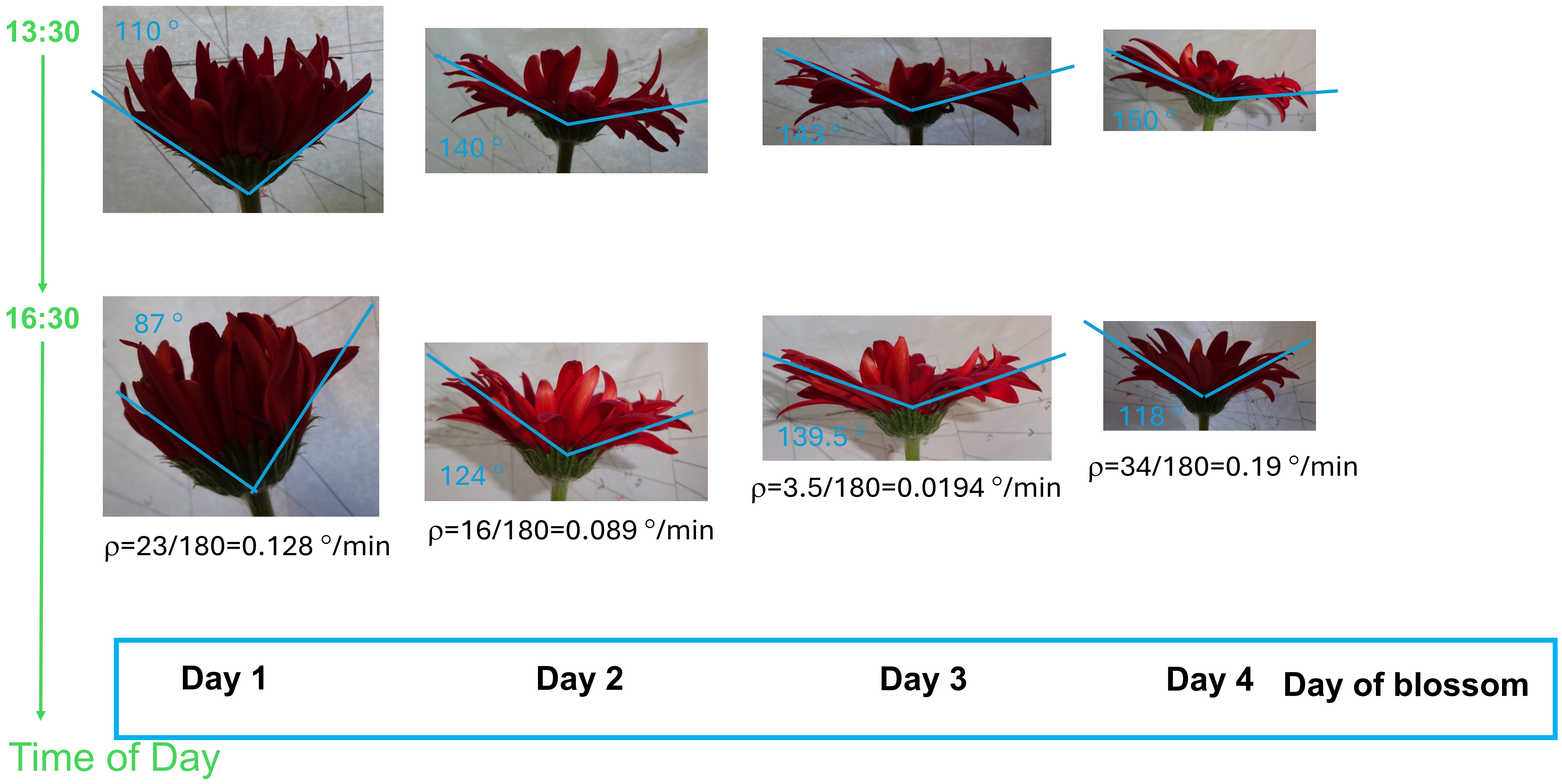

Dates: 7/7-10/24, Flower: 50-C-w

13:30

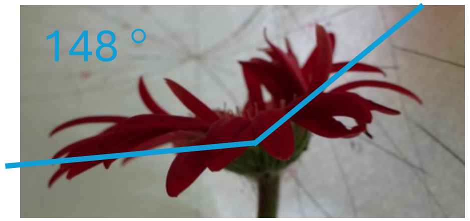

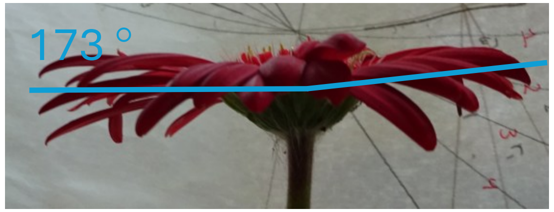

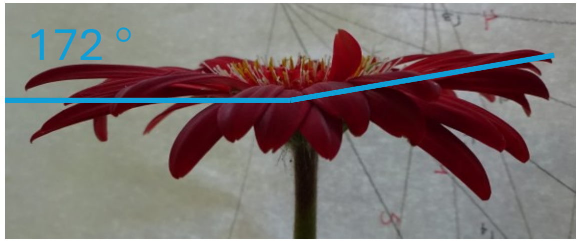

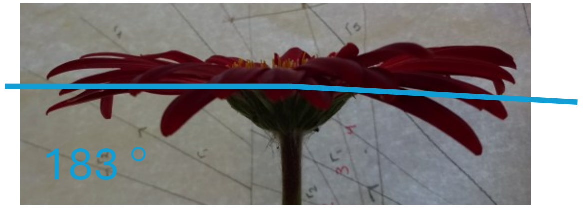

16:30

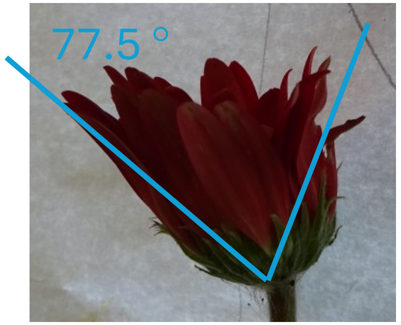

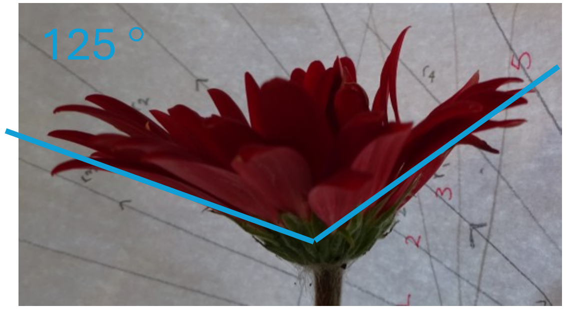

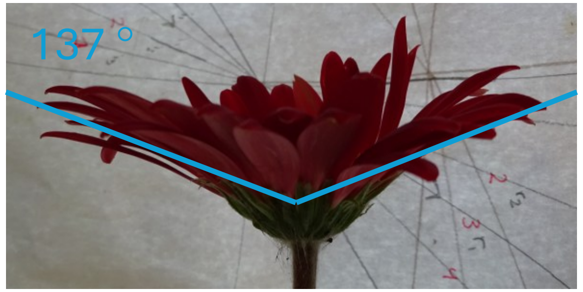

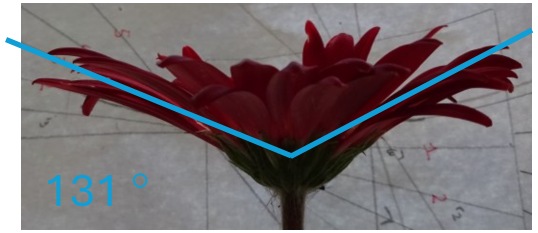

ρ=70.5/180=0.392 °/min

ρ=48/180=0.27 °/min

ρ=35/180=0.194 °/min

ρ=52/180=0.29 °/min

Day 1 **Day 2** **Day 3** **Day 4**

Day of blossom

Time of Day

Dates: 7/7-10/24, Flower: 50-D-w

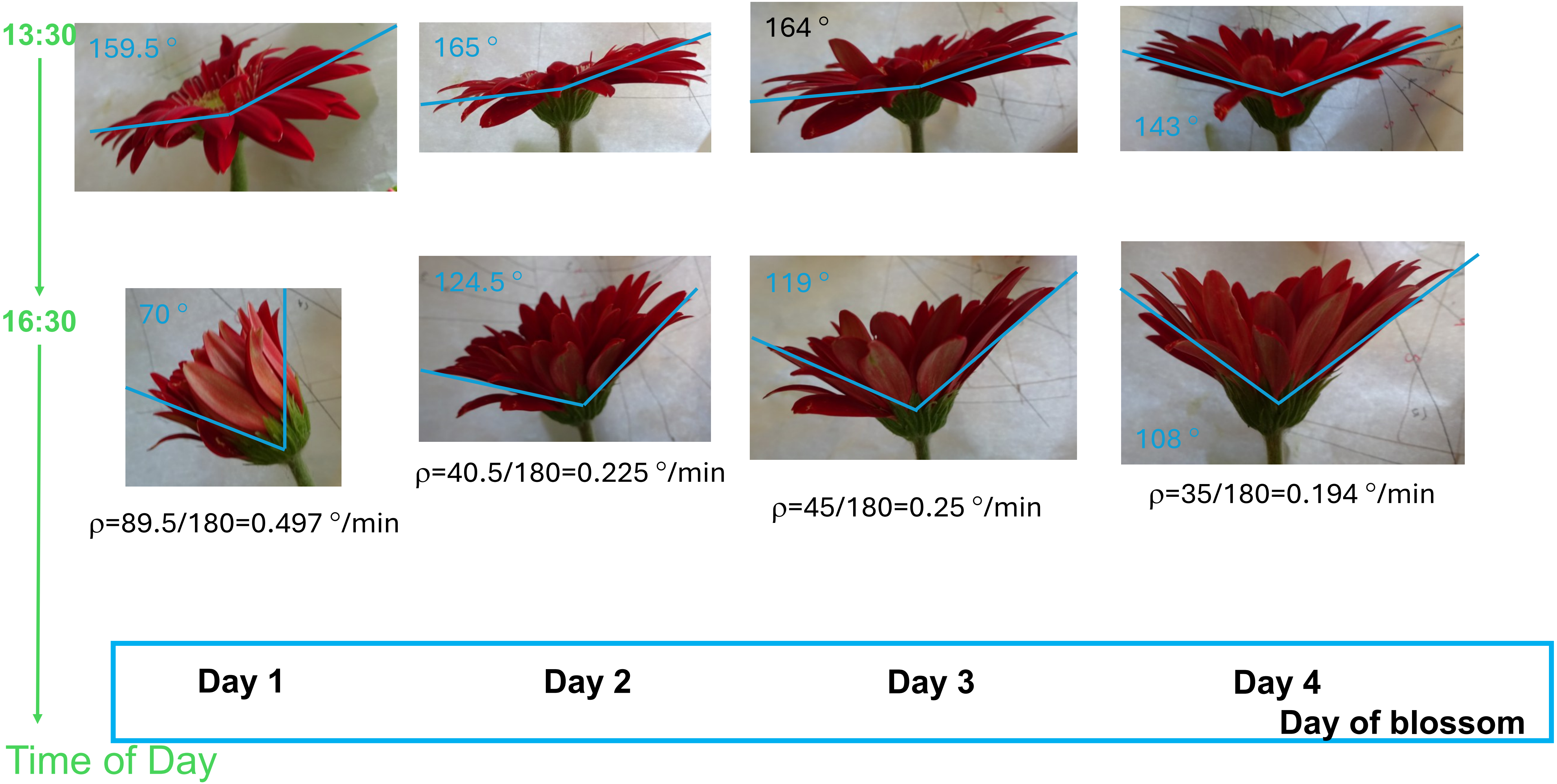

Dates: 7/15-17/24, Flower: 51-A-w

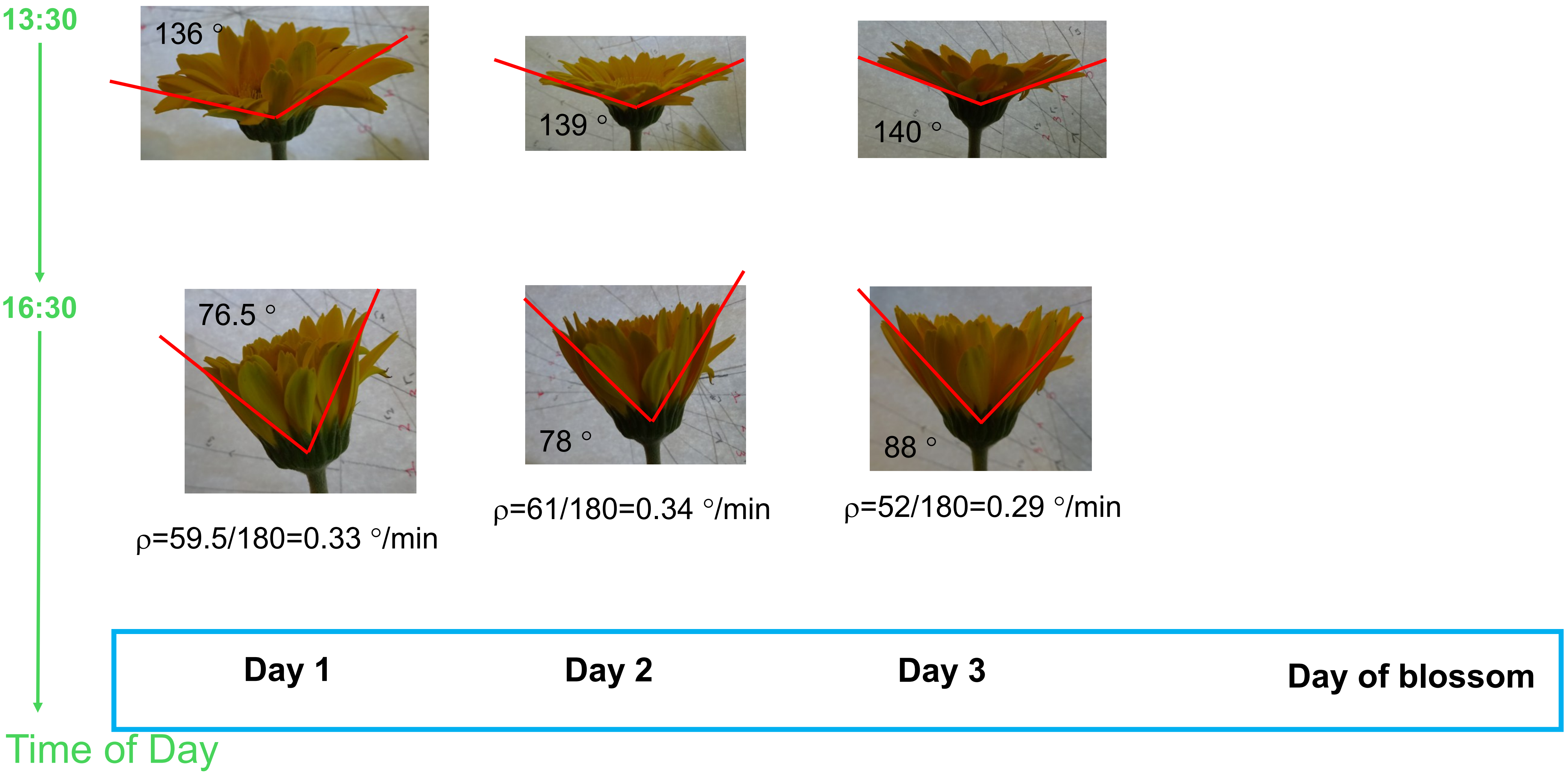

Supplement: S1 Dataset — This file contains tables and images with the raw data that enabled extracting the results for the rate of closure of G. jamesonii presented in this work. (PDF) [file pone.0343498.s002.pdf]
