# Supplementary material for: A phenomenological comparison of the effects of blue light, red light and radio waves on the escape speed of Caenorhabditis elegans and the rate of closure of Gerbera jamesonii petals
Source: PLoS One. 2026 Apr 1;21(4):e0343498. doi: 10.1371/journal.pone.0343498 (PMC13043045; doi:10.1371/journal.pone.0343498)
Supplement: S1 Text — This file contains additional information on the shielding effect of the EPS enclosures, on the classic electromagnetic waves energy in scientific literature, and on the accuracy of the Random Forest Classifier. (DOCX) [file pone.0343498.s003.docx]

**A phenomenological comparison of the effects of blue light, red light and radio waves on the escape speed of *Caenorhabditis elegans* and the rate of closure of *Gerbera jamesonii* petals**

Alexander W. Kline^1¶^, Charles S. Beattie^1¶^, Addison K. Shenk^2&^, Samuel I. Spicher^2&^, Timothy A. Bloss^3^, Laura Tipton^3,4^, Marquis T. Walker^3^, Laura G. Vallier^5^, Kristopher L. Schmidt^2^*, and Giovanna Scarel^1^*

^1^Department of Physics and Astronomy, James Madison University, Harrisonburg, VA, United States of America

^2^Department of Biology and Chemistry, Eastern Mennonite University, Harrisonburg, VA, United States of America

^3^Department of Biology, James Madison University, Harrisonburg, VA, United States of America

^4^Department of Mathematics & Statistics, James Madison University, Harrisonburg, VA, United States of America

^5^Department of Biology, Hofstra University, Hempstead, NY, United States of America

*Corresponding Authors

E-mail: [scarelgx@jmu.edu](mailto:scarelgx@jmu.edu) (GS), [kristopher.schmidt@emu.edu](mailto:kristopher.schmidt@emu.edu) (KS)

^¶^ These authors contributed equally to this work

^&^ These authors also contributed equally to this work

**Short title**: Comparison of the effects of light and radio waves on biological organisms

**Index:**

1. **The shielding effect of the EPS enclosures p. 2**
2. **The classic electromagnetic waves energy in scientific literature p. 4**

**(a) Propulsion of nanoparticles p. 5**

**(b) Rotation of nanoparticles induced by laser light p. 7**

**(c) Fast molecular dynamics p. 15**

# (d) Photodetector physics p. 16

1. **Accuracy of Random Forest Classifier p. 18**
2. **The shielding effect of the EPS enclosures**

In all our experiments we placed *C. elegans* and *G. jamesonii* into expanded polystyrene (EPS) enclosures. The EPS enclosures shielded from external disturbances the biological organisms and the sources we used to generate electromagnetic waves. The shielding effectiveness of the EPS enclosures is well documented in S1 Fig, in which we compared the voltage produced in time by a 3TECBT detector illuminated with radio waves. The 3TECBT detectors were described in Ref. 1. We performed the voltage measurements with the EPS enclosures in a dark room to avoid false effects due to either natural or artificial light. To ensure electrical, thermal and noise insulation, we placed detectors, antennas and electromagnetic waves sources within the EPS enclosure. The distance between detector and source was 15-20 cm.


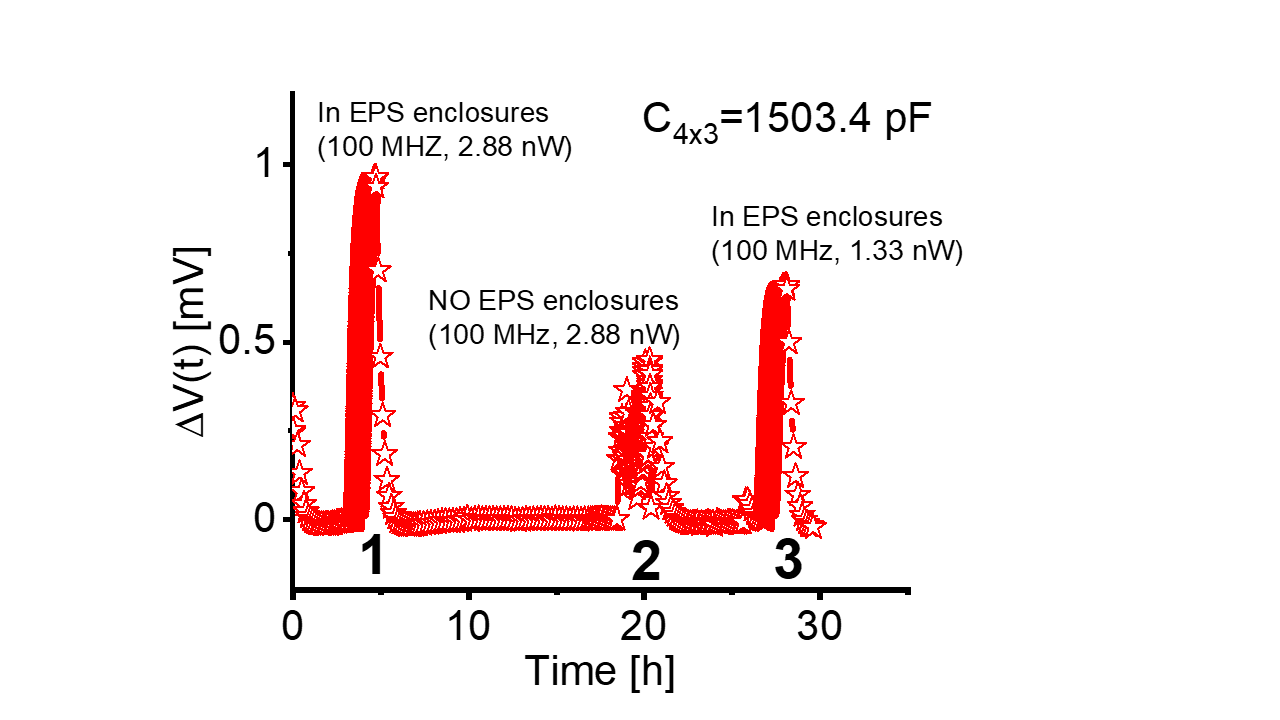


**S1 Fig. Voltage difference ΔV(t) versus time t (in hours) collected from a 3TECBT detector illuminated with 100 MHz radio waves.** The feature labeled “1” was collected with detector, source and antenna within an EPS enclosure and radio waves at 2.88 nW. The feature labeled “2” was collected with the same radio waves, but with detector, source and antenna without EPS enclosure. The feature labeled “3” was collected with detector, source and antenna inside the EPS enclosure, but with 100 MHz radio waves at 1.33 nW. The detector had capacitance C_cap_=1503.4 pF.

In S1 Fig we reported the voltages produced in time by a 3TECBT detector illuminated with 100 MHz radio waves at 2.88 nW. When placed inside the EPS enclosure, the detector generated the neat exponentially increasing feature labeled “1” with ΔV~1.1 mV. However, without the shield of the EPS enclosure, the detector produced only the faint and blurry feature labeled “2”. When illuminated with 100 MHz radio waves at 1.33 nW, the detector also produced an exponentially increasing feature labeled “3” with ΔV~0.7 mV. These data are as reported in the Supplementary Materials of Ref. 1. From these data we inferred that the environmental disturbances caused the blurry feature labeled “2” and because of this finding we opted to perform all our experiments with biological organisms inside the EPS enclosures.

1. **The classic electromagnetic waves energy in scientific literature**

The derivation of Larmor’s equation leading to the classic electromagnetic wave energy is outlined in the textbooks by D. Griffiths and J. Jackson [2,3]. A detailed step-by-step derivation of Larmor’s equation in classical electrodynamics is presented in the class notes for the course PHYS 350 “Electricity and Magnetism” at JMU found in <https://github.com/Charlesbt12/Supplementary-1-Maxwell-to-Larmor-PLOS-one-350>.

Here we prove the effectiveness of the electromagnetic wave energy in interpreting data published in scientific literature. The findings motivated us adopting the electromagnetic wave energy as the energy transferred or emitted in the interactions between electromagnetic waves and matter, including biological organisms. In the cases reported below we expressed the classic electromagnetic wave energy as Pτ, the product of the power P with the period τ, which is the inverse of the frequency ν such that τ=$\frac{1}{}$. This expression is equivalent to that used in the Main Text, i.e.$\frac{P}{}$.

1. **Propulsion of nanoparticles**

The propulsion of optically transparent spherical silica Janus nanoparticles (SSJN) with propulsion velocity v_p_ described in C. Lozano *et al*. [4], provides a framework in which Pτ is the energy transferred to a detector with λ<l_act_, so that the effective power is P_λ_=P$\frac{}{l_{act}}$, where l_act_ is the one-dimensional size of the detector and $\frac{}{l_{act}}$ the *antenna factor*. C. Lozano *et al*. [4] adopt SSJNs with r=1.35 μm, and cap them on one side with a thin light-absorbing carbon layer. The authors then suspend the SSJNs in a binary critical mixture of water-2,6-lutidine (wl), and put them in motion with laser light at λ=532 nm (ν=0.565 PHz, and τ=1.77 fs). C. Lozano *et al*. [4], in their Figure 2(c), find that, in a quasi-static illumination landscape and above the threshold intensity I_t_=0.25 $\frac{\mu W}{{\mu m}^{2}}$, the v_p_ of the SSJNs is only determined by the local intensity incident on the particle, not by the intensity gradient ∇I. Moreover, the authors show that v_p_ increases almost linearly with light’s intensity I. From Figure 2(c) of Ref. 4, we infer that v_p_=5 $\frac{\mu m}{s}$ is achieved at I=0.65 $\frac{\mu W}{{\mu m}^{2}}$. Assuming the laser light provides the SSJNs with homogeneous illumination on their 22.9 (μm)^2^ surface area, we infer that the average power P_ave_ on each SSJN is ~(0.65/22.9) μW=0.02838 μW=28.38 nW. Thus, the effective power P_λ_=P_ave_*λ/2r is 5.59 nW. This result reflects the fact that λ=532 nm<2r=2700 nm. Energy conservation in the interaction between the laser light and a SSJN immersed in wl, gives us:

P_λ_τ=½m_J_v_p_^2^+η_wl_*m_wl_/Δt (S1)

The first addend accounts for the light-activated self-propulsion of the SSJNs. The second addend captures the effect on the environment (the wl) of the irradiation and of the SSJN’s motion. Thus, Eq. (S1) enables us to extract and discuss the kinematic viscosity η_wl_ of the wl in which the SSJNs move. If we obtain reasonable η_wl_ values, then we may infer that we can be confident that the energy P_λ_τ is transferred from the light to the SSJNs. To this end, we estimate the parameters in Eq. (S1). First, we determine m_J_, the mass of each SSJN, as m_J_=$\frac{\delta_{sil}}{V_{J}}$, where δ_sil_=2.65 10^3^ $\frac{kg}{m^{3}}$ is the density of silica, and V_J_=10.31 10^-18^ m^3^ is the volume of each SSJN. We find m_J_=27.32 10^-15^ kg. Then, we determine m_wl_, the mass of the wl displaced by a SSJN, as m_wl_=$\frac{\delta_{wl}}{V_{wl}}$, where δ_wl_=0.92 10^3^ $\frac{kg}{m^{3}}$ is the density of the wl [5], and V_wl_=10.31 10^-18^ m^3^ is the wl volume displaced by each SSJN. We find m_wl_=9.48 10^-15^ kg. Finally, we evaluate Δt assumed as the time interval in which the probability distribution P(x) of self-propelled SSJNs is defined (see Figure 1(a) of Ref. 4). For Δt=5 s, P(x) is narrowly spread in space, and the SSJN are in the reorientation phase, as we infer from Figure 3(b) of Ref. 4. In this case, η_wl_=0.005 $\frac{{mm}^{2}}{s}$. This value is three orders of magnitude smaller than η_H2O_=1 $\frac{{mm}^{2}}{s}$, the kinematic viscosity of water, which implies that the SSJNs effect on the resistive flow of the wl is negligible. For Δt=500 s, P(x) starts broadening in space, as seen in Fig. 1(a) [4]. In this case, η_wl_=0.5 $\frac{{mm}^{2}}{s}$, which is $\frac{1}{2}$of η_H2O_=1 $\frac{{mm}^{2}}{s}$. Such value suggests that the SSJNs effect on the resistive flow of the wl is still low. For Δt=2500 s, P(x) further broadens in space, as reported in Fig. 1(a) [4]. In this case, η_wl_=2.52 $\frac{{mm}^{2}}{s}$, which is more than twice η_H2O_=1 $\frac{{mm}^{2}}{s}$. This fact signals that the SSJNs dramatically increase the resistive flow of the wl. Thus, from data in Ref. 4, we conclude that, in the presence of the SSJNs, the wl acquires rheopectic behavior, which means that the kinematic viscosity increases with time. In other words, the longer the wl undergoes shearing force, the higher its viscosity. Examples of rheopectic fluids include some lubricants, [gypsum](https://en.wikipedia.org/wiki/Gypsum) pastes, printer [inks](https://en.wikipedia.org/wiki/Ink), and the [synovial fluid](https://en.wikipedia.org/wiki/Synovial_fluid) in the human body.

1. **Rotation of nanoparticles induced by laser light**

Another case in which Pτ proves to be the energy transferred to a detector is the rotation of silica dumbbell nanoparticles (SDNs) induced in vacuum by laser light described in Refs. 6, 7-9. The authors describe SDNs as nanoparticles consisting of two spheres, each of radius R, mass m_0_, volume V, and density δ. R. Reimann *et al*. [6, 7-9] induce the rotation of SDNs using near infrared (NIR) laser light with wavelength λ=1565 nm (frequency ν=0.192 PHz, period τ=5.21 fs) at constant trapping power P_trapp_=226 mW. On the other hand, J. Ahn *et al*. [9] adopt tightly focused NIR laser light with λ=1550 nm (ν=0.194 PHz, τ=5.17 fs) at P_trapp_=500 mW. Both teams perform experiments in a vacuum chamber at a gas pressure p_gas_ ranging from 7.2 10^-4^ Pa to 1.333 10^-1^ Pa, as shown in S1and S2 Tables [6, 7-9], along with the corresponding rotation frequencies f_rot_ achieved by the SNDs.

**S1 Table. Environmental and mechanical parameters for the SDNs from Ref. 6.**

| **p_gas_ (mbar)** | **p_gas_ (Pa)** | **f_rot_ (Hz)** | **f_rot_ (GHz)** | **ξ** | **m_eff_=m_0_*ξ**  **(10^-18^ kg)** |
| --- | --- | --- | --- | --- | --- |
| 1*10^-4^ | 1 10^-2^ | 6 10^8^ | 0.6 | 0.937 | 0.937 |
| 1.1*10^-5^ | 1.1 10^-3^ | 6.55 10^8^ | 0.655 | 0.336 | 0.336 |
| 1*10^-5^ | 1 10^-3^ | 8.5 10^8^ | 0.85 | 0.278 | 0.278 |
| 7.2*10^-6^ | 7.2 10^-4^ | 1.029 10^9^ | 1.029 | 0.144 | 0.144 |

Pressure in the vacuum chamber p_gas_ in mbar and Pa, rotation frequency f_rot_ in Hz and GHz, correction factor ξ and effective mass m_eff_ of the SDNs from Ref. 6.

**S2 Table. Environmental and mechanical parameters for the SDNs from Ref. 9.**

| **p_gas_ (torr)** | **p_gas_ (Pa)** | **f_rot_ (Hz)** | **f_rot_ (GHz)** | **ξ** | **m_eff_=m_0_*ξ**  **(10^-18^ kg)** |
| --- | --- | --- | --- | --- | --- |
| 1*10^-3^ | 1.333 10^-1^ | 1.1 10^8^ | 0.11 | 4271.1 | 5979.5 |
| 1.6*10^-4^ | 2.1 10^-2^ | 5 10^8^ | 0.5 | 5.95 | 8.33 |
| 1*10^-4^ | 1.333 10^-2^ | 9 10^8^ | 0.9 | 1.836 | 2.57 |

Pressure in the vacuum chamber p_gas_ in torr and Pa, rotation frequency f_rot_ in Hz and GHz, correction factor ξ and effective mass m_eff_ of the SDNs from Ref. 9.

Both teams observe that f_rot_∝$\frac{1}{p_{gas}}$, as documented in Figure 2(b) [6] and Figure 4(c) [9]. We summarize the observed relationship between f_rot_ and p_gas_ in S1 Fig. Here, utilizing data in Refs. 6 and 9, we find that f_rot_~0.0011 GHz*Pa ($\frac{1}{p_{gas}}$) and f_rot_~0.013 GHz*Pa ($\frac{1}{p_{gas}}$), respectively. The observed relationship between f_rot_ and p_gas_ is explained as the result of the variations in the applied torque T [6,9].


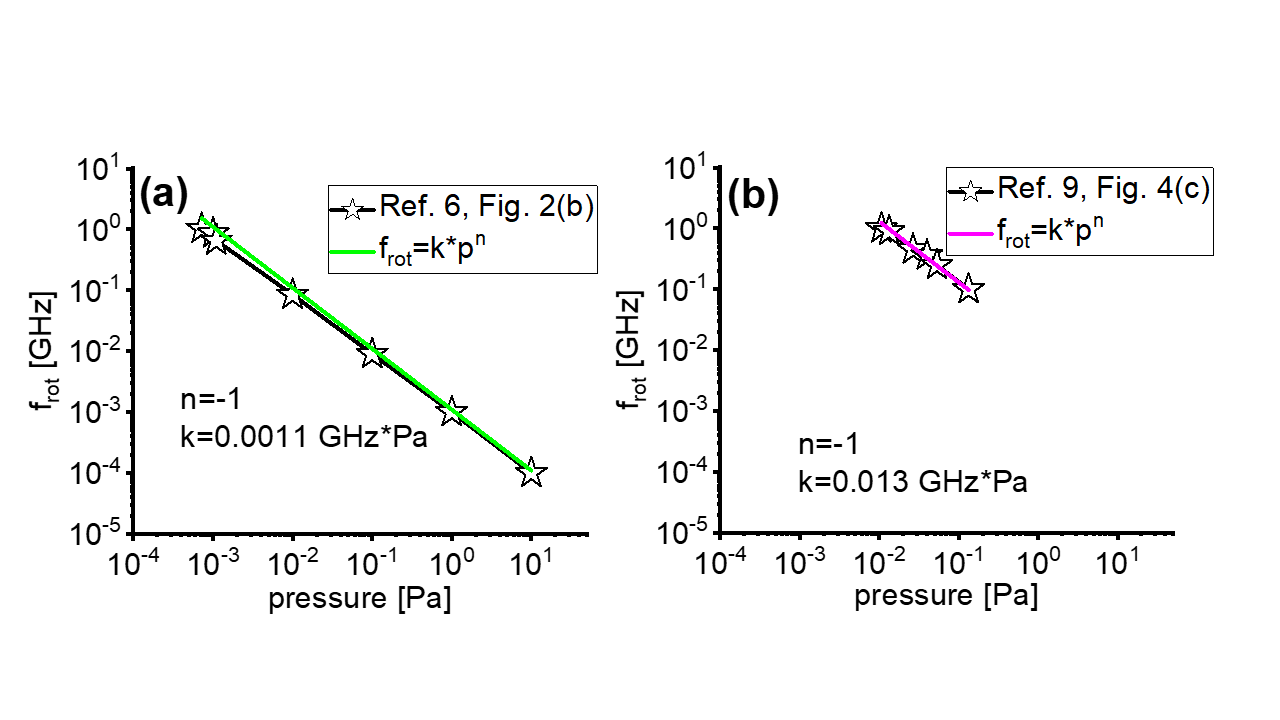


**S2 Fig. Relationship between rotation frequency f_rot_ and pressure p_gas_ in the vacuum chamber**. In panel (a) the data were derived from Figure 2(b) of Ref. 6. In panel (b) the data were derived from Figure 4(c) of Ref. 9. The value of n=-1 was found in agreement with Refs. 6 and 9. The values of k were determined from our analysis.

Explicitly determining the relationship among T, f_rot_, p_gas_, and the laser beam’s energy P_trapp_τ requires knowing the details of SDNs and illumination conditions. References 6, 7-9 report R≅50 nm, m_0_=1 fg [6,8] and ~1.4 fg [9], V≅0.52 10^-15^ cm^3^, and either δ=2.22 $\frac{g}{{cm}^{3}}$ [7], or 2.65 $\frac{g}{{cm}^{3}}$ [9]. For f_rot_, both teams report the maximum values achieved by selecting the quarter-wave-plate angle ϕ where the laser light is nearly-left-circularly-polarized. This angle is -29° in Ref. 7, whereas it is not specified in Ref. 9. By fixing P_trapp_, λ, and ϕ, the laser beam secures constant polarizability to the SDN’s rotation [7-10]. In these experiments the ratio N=$\frac{{}_{ex}}{l_{act}}$=$\frac{1565 nm}{50 nm}$=31.3 is the *antenna factor*.

According to R. Reimann *et al*. [6], the torque T involved in the SDN’s rotation is expressed in their Equation (1) [6]:

$\frac{d}{\mathrm{dt}}$L=I$\frac{d}{\mathrm{dt}}$f_rot_=T_opt_+T_drag_,

or

$\frac{1}{2}$If_rot_^2^=T_opt_+T_drag_.

The $\frac{1}{2}$If_rot_^2^ term is obtained upon integration of I$\frac{d}{\mathrm{dt}}$f_rot_ over a range of frequencies. The parameter L is the SDN’s angular momentum, whereas I=2.8 m_0_ R^2^ is its moment of inertia [8]. The torque T_drag_ results from the effects of the environment. On the other hand, T_opt_ depends on the laser light and its interaction with the SDNs. Let us separately analyze T_drag_ and T_opt_.

The torque T_drag_=If_rot_/t_damp_, where t_damp_=β m_0_ v_mol_/(p_gas_R^2^) is the damping time [6]. Thus:

T_drag_=(If_rot_p_gas_R^2^)/(βm_0_v_mol_)

=Γf_rot_p_gas_

Here, Γ=(IR^2^/m_0_)/(βv_mol_)=17.5 10^-30^ m^4^/(βv_mol_).

Assuming the accommodation factor β=0.2 [10], the mean molecular velocity of the N_2_ molecules in the vacuum chamber v_mol_≅300 $\frac{m}{s}$, and p_gas_=1.1 10^-3^ Pa (Figure 2(b) [6]), we obtain t_damp_≅22 s [10]. Moreover, we find that, at p_gas_=1.1 10^-3^ Pa and f_rot_≅0.628 GHz (Figure 2(b) [6]):

T_drag_=0.2 10^-24^ Nm (or J) [10].

The torque T_opt_=T_abs_+T_brf_+T_shape_ results from the contribution of three addends [6]. The first is:

T_abs_=$\frac{}{2c}$σ_abs_Δs_abs_(IN)

=$\frac{1}{2A}$(σ_abs_Δs_abs_)P_trapp_τ,

where IN=P_trapp_/A is the laser beam’s intensity, and A is the illuminated area which we assume to correspond to half of a spherical surface for each sphere in the SDN, i.e. A=4πR^2^. The degree of circular polarization Δs_abs_ is selected to be |1| [6], which corresponds to the nearly-left-circularly-polarized laser light, where f_rot_ achieves its maximum value [6]. The parameter σ_abs_ is the absorption cross section of the SDNs, whose magnitude is unknown. However, we can estimate its minimum value σ_abs-min_ by constraining T_abs_ to be of the order of the previously found T_drag_, i.e. 0.2 10^-24^ Nm. With this constraint and using P_trapp_=226 mW and τ=5.21 fs [6], we obtain σ_abs-min_~33 10^-24^ m^2^. This value is reasonable, because it is of the order of values found in the literature (e.g. CdS nanocrystals have σ_abs_=17 10^-24^ m^2^, see the Supplementary Materials on page 14 of Ref. 11). By adopting σ_abs-min_~33 10^-24^ m^2^, the minimum value of T_abs_ is $\frac{1}{2}$1.05 10^-9^ P_trapp_τ, which has units of Nm (or J).

The second addend in T_opt_ is T_brf_. M. Friese *et al*. [12], cited in Ref. 6, propose:

T_brf_=$\frac{c}{2}$E_0_^2^ΠA

where Π is the sum of terms containing products of sine and cosine functions of the optical parameters of the laser light [13], such that 0≤|Π|≤1. Using Poynting’s vector, i.e. IN=$\frac{c}{2}$|E_0_^2^|, we obtain:

T_brf_=$\frac{}{}$P_trapp_

=$\frac{}{2}$P_trapp_τ.

We can estimate minimum value Π_min_ by constraining T_brf_ to be of the order of the previously found T_drag_, i.e. 0.2 10^-24^ Nm. With this constraint and using P_trapp_=226 mW and τ=5.21 fs [6], we obtain Π_min_~1.07 10^-9^. By adopting Π_min_, the minimum value of T_brf_ is $\frac{1}{2}$ 1.07 10^-9^ P_trapp_τ and has units of Nm (or J).

The third addend in T_opt_ is T_shape_. S. Kuhn *et al*. [14], cited in Ref. 6, propose in their Eq. (2):

T_shape_=$\frac{l^{2}d^{4}}{48w^{2}}$P_trapp_$\frac{k^{3}}{c}$[Δχη_1_(kl)+χ_⊥_η_2_(kl)]

=ςP_trapp_τ.

In this equation, Δχ is the susceptibility anisotropy, k=$\frac{1}{}$ is the wave vector, c is the speed of light, and w is the light’s beam waist radius, which is the only unknown parameter. Adapting the parameters l and d to the SDNs of Ref. 6, we have l~2R, d~2R. Since in Ref. 6 R/λ=0.03<<1, then, according to Ref. 14, η_1_(kl)≅1 and η_2_(kl)=0, thus Δχη_1_(kl)+χ_⊥_η_2_(kl)=Δχ [14]. Since Δχ=(ε_r_-1)^2^/(ε_r_+1), and the relative dielectric constant of silica is ε_r_≅3, we conclude that Δχ≅1. Therefore, ς=$\frac{4{}^{2}R^{6}}{3{}^{4}w^{2}}$≅$\frac{4R^{6}}{3{}^{4}w^{2}}$. We can estimate minimum value ς_min_ by constraining T_shape_ to be of the order of the previously determined T_drag_, i.e. 0.2 10^-24^ Nm. With this constraint and using P_trapp_=226 mW and τ=5.21 fs [6], we obtain ς_min_~0.17 10^-9^. By adopting ς_min_, we estimate the minimum beam’s waist radius w_min_ to be 4.5 μm. This value is in a reasonable range, being slightly smaller than 27 μm, the beam waist radius cited in Ref. 14. Referring to the experiment of R. Reimann *et al*. [6], and F. van der Laan *et al*. [8], a w_min_≅4.5 μm would be able to trap in the laser beam focus a maximum of 45 and 18 SDNs composed of spheres with diameter ranging from 100 nm and 250 nm, respectively. By adopting ς_min_, the minimum value of T_shape_ is 0.17 10^-9^ P_trapp_τ and has units of Nm (or J).

In summary,

$\frac{1}{2}$If_rot_^2^=$\frac{1}{2A}$(σ_abs_Δs_abs_)P_trapp_τ+$\frac{}{2}$P_trapp_τ+ςP_trapp_τ+Γf_rot_p_gas_

={$\frac{1}{2A}$(σ_abs_Δs_abs_)+$\frac{}{2}$+ς}P_trapp_τ+Γf_rot_p_gas_. (S2)

We can further simplify Eq. (S2) by considering the experimental relationship between f_rot_ and p_gas_ emerged from data in Ref. 6 and summarized in S2 Fig. Here, we found f_rot_~0.0011 GHz*Pa ($\frac{1}{p_{gas}}$). Thus, we can further write T_drag_=Γf_rot_p_gas_=19.25 10^-24^ $\frac{Nm^{2}}{s}$*(1/β*v_mol_). This expression gives β*v_mol_=96.25 $\frac{m}{s}$ for T_drag_= 0.2 10^-24^ Nm, in good agreement with the values we assumed above (i.e. β=0.2, v_mol_=300 $\frac{m}{s}$, and β*v_mol_=60 $\frac{m}{s}$). Thus, we can further summarize:

$\frac{1}{2}$If_rot_^2^={$\frac{1}{2A}$(σ_abs_Δs_abs_)+$\frac{}{2}$+ς}P_trapp_τ+19.25 10^-24^ $\frac{Nm^{2}}{s}$*(1/β*v_mol_). (S3)

Equation (S3) shows that P_trapp_, τ, β, and v_mol_ are the main variables determining f_rot_ with nearly-left-circularly-polarized laser light.

Alternatively, we explain the observed relationship between f_rot_ and p_gas_ as resulting from the variations in the energy transferred from the trapping laser light to the SDNs. We hypothesize this energy to be P_trapp_τ=$\frac{1}{2}$I_eff_f_rot_^2^. In this equation, I_eff_=2.8 m_eff_ R^2^ is the moment of inertia of the SDN [8], and m_eff_=m_0_*ξ its effective mass. We introduce m_eff_, and thus the dimensionless factor ξ, to enable changes in p_gas_ to induce changes in f_rot_. The dimensionless factor ξ=$\frac{m_{eff}}{m_{0}}$ represents the ratio between the effective (m_eff_) and the actual SDN’s mass m_0_. The need of introducing m_eff_ arises because the decreasing gas pressure affects the SDN’s mass as if “reducing” it. Such effect resembles that of the increasing distance d_E_ of an object from Earth’s surface, which decreases the gravitational acceleration |**g**| as $\frac{1}{d_{E}^{2}}$, and, consequently, the object’s weight. One main difference is that, while |**g**| has units ($\frac{m}{m^{2}}$), ξ is dimensionless. We summarize the values of p_gas_, f_rot_, ξ and m_eff_ in S1 Table for data from Ref. 6 and in S2 Table for data from Ref. 9. We show the relationship between ξ and p_gas_ in S3 Fig with data from Refs. 6 and 9. From S1 and S2 Tables**,** and S3 Fig we infer that, when p_gas_ decreases, so do also ξ and m_eff_. At the same time, f_rot_ increases to keep the energy P_trapp_τ constant (law of conservation of energy). Although the data were collected at lower p_gas_ in Ref. 6 than in Ref. 9, with slightly different P_trapp_ and τ, the overall ξ versus p_gas_ trend can be fitted with the exponential function:

ξ=ξ_0_ exp^(p_gas_/p_gas_*)

where ξ_0_=0.3, and p_gas_*=1.333 10^-2^ Pa. We conclude that the ability of the function ξ=ξ(p_gas_) to account for data sets from different teams testifies the validity of our approach with P_trapp_τ as the energy transferred from laser to SDN.

We also notice that P_trapp_τ=$\frac{1}{2}$I_eff_f_rot_^2^=ξ$\frac{1}{2}$If_rot_^2^. Thus, from our Eqs. (S2) and (S3), we infer that:

ξ≅{$\frac{1}{2A}$(σ_abs_Δs_abs_)+$\frac{}{2}$+ς}^-1^.


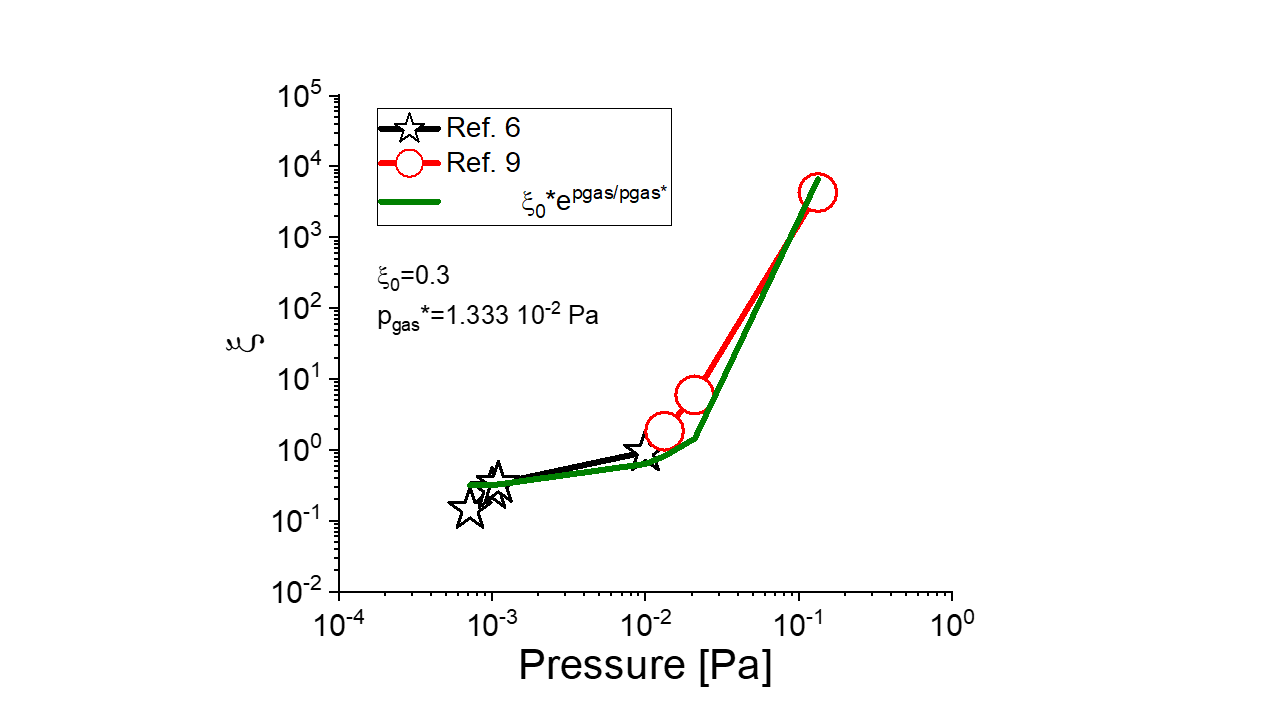


**S3 Fig. Dimensionless factor ξ versus gas pressure p_gas_ in the vacuum chamber.** The data point were derived from Refs. 6 and 9. In our analysis we used the fitting function ξ=ξ_0_ exp^(p_gas_/p_gas_*) where ξ_0_=0.3 and the pressure constant was fixed at p_gas_*=1.333 10^-2^ Pa.

1. **Fast molecular dynamics**

Two examples of the effectiveness of E_EM_=Pτ in accounting for the energy transferred from electromagnetic (EM) waves to matter come from the dynamics of biological phenomena captured by time resolved serial femtosecond X-ray (TR-SFX) crystallography technique at the Swiss Free Electron Laser (SwissFEL) source. The first is the dynamics of a light-driven chloride pump [15]. S. Mous *et al*. [15] study a photoactive chloride-pumping halo-rhodopsin from the marine bacterium known as *Nonlabens marinus* (*Nm*HR). To initiate transport, the retinal chromophore within the *Nm*HR needs to be involved. The retinal chromophore has a Cl^351^ binding position in the resting (or dark) state. This binding position needs to be transformed into a new Cl^352^ binding position to promote chloride transport. The transformation from the Cl^351^ to the Cl^352^ binding position occurs through a photo-induced configurational change from *all-trans* to *13-cis* of the retinal chromophore. The details of how this mechanism initiates the chloride transport cycle are not understood; however, the events of the transport follow a modified Jardetzky access model [15, 16]. S. Mous *et al*. [15] activate the retinal chromophore with green pump laser light at λ=530 nm (ν=0.566 PHz, τ=1.767 fs) [17] at repetition rate r=50 Hz and pulse energy E_p_=2.5 μJ (see Supplemental Materials in Ref. 15). The average power of the laser is thus P_ave_=r*E_p_=0.125 mW. The authors observe that, 10 ps after the beginning the illumination, a hydrogen bond breaks, giving rise to charge separation between the protonated Schiff base (PSB) and the chloride binding site Cl^351^ in the retinal chromophore. Using [quantum mechanics](https://en.wikipedia.org/wiki/Quantum_mechanics)/[molecular mechanics](https://en.wikipedia.org/wiki/Molecular_mechanics) (QM/MM) [simulation](https://en.wikipedia.org/wiki/Simulation)s, S Mous *et al*. [15] estimate that, in this charge separation process, an energy of 28.2 $\frac{kcal}{mol}$ is stored in the retinal chromophore. This energy corresponds to 0.196 aJ=1.225 eV per retinal chromophore. Assuming Pτ as the energy transferred from the green pump laser light at λ=530 nm to the retinal chromophore, we estimate that P_ave_=0.111 mW can supply such energy (i.e. 0.196 aJ=1.225 eV per retinal chromophore). We notice that P_ave_=0.111 mW is very close to P_ave_= 0.125 mW, the average power of the pulsed laser experimentally used to activate the retinal chromophore. The excellent agreement between the experimental and the estimated value of P_ave_ supports the validity of the hypothesis that Pτ is the energy transferred from the EM waves to the retinal chromophore.

The second example consists of the dynamics of light-driven deoxyribonucleic acid (DNA) repair [18]. In Ref. 18, N. Christou *et al*. use a pump laser at λ=396 nm (0.757 PHz, 1.32 fs) which focuses with a peak energy of E_p_=10 μJ on the sample with a repetition rate r=100 Hz, and pulse duration of δ=1.32 ps. From the information given, we determine that the peak power on the sample is P_peak_=$\frac{E_{p}}{\delta}$=7.57 MW, whereas the average power P_ave_=E_p_*r=1 mW. This finding means that the energy transferred from the light to the DNA is P_ave_τ=1.32 aJ=8.25 eV. This value is of the same order of magnitude as that in the experiment in Ref. 15, and of the same order of the typical energy to activate chemical processes. Finally, we observe that the P_ave_ values of either 0.111 mW or 0.125 mW in Ref. 15 and P_ave_≅1 mW in Ref. 18 are of the order of magnitude of 0.15 $\frac{mW}{{mm}^{2}}$ used in Ref. 19 for experiments on light-gated channelrhodopsin.

# Photodetector physics

A final example of the successful application of the energy Pτ is from photodetector physics. For example, E. Rowe *et al*. [20] study photo-induced room temperature superconductivity in an organic molecular solid crystal of K_3_C_60_. They compare the photoexcitation of this material from 100 K to room temperature with middle infrared (MIR) light at 170 meV (ν=41.02 THz, λ=7.31 μm, τ=24.38 fs) with fluence 18 $\frac{mJ}{{cm}^{2}}$, and at 41 meV (ν=9.894 THz, λ=30.32 μm, τ=101.07 fs) with fluence 0.4 $\frac{mJ}{{cm}^{2}}$. The photoexcitation of K_3_C_60_ is provided by lasers at repetition rate R=100 MHz (stated for the 170 meV case, assumed for the 41 meV case), which enables us to estimate the average power P_ave_ in the two cases: for the 170 meV case P_ave_=1800 $\frac{kW}{{cm}^{2}}$, whereas for the 41 meV case P_ave_=40 $\frac{kW}{{cm}^{2}}$. The energy E_EM_=Pτ is 43.9 $\frac{nJ}{{cm}^{2}}$ for the 170 meV case, and 4.04 $\frac{nJ}{{cm}^{2}}$ for the 41 meV case. If we consider that, assuming l_act_=1 μm, for the 170 meV case P_λave_=13.16 $\frac{MW}{{cm}^{2}}$, and for the 41 meV case P_λave_=1.21 $\frac{MW}{{cm}^{2}}$, then the P_λ_τ energies are 320.8 nJ and 122.6 nJ, respectively. So, we may safely conclude that the energies transferred from the 170 meV and the 41 meV lasers are of the same order of magnitude. E. Rowe *et al*. [17] detect the surge of superconductivity from the behavior of the reflectivity R(ω), and the real and imaginary parts, σ_1_(ω) and σ_2_(ω), respectively, of the optical conductivity. They observe that R(ω), σ_1_(ω) and σ_2_(ω) are very similar after excitations at 170 meV and 41 meV, despite the two orders of magnitude difference of the fluences in the two cases (18 $\frac{mJ}{{cm}^{2}}$, and 0.4 $\frac{mJ}{{cm}^{2}}$, respectively). E. Rowe *et al*. [17] ascribe their result to the excitation of the IR-active T_1u_ phonon in K_3_C_60_, not to the similar energy injected in the system in the two cases. Here, the E_EM_=Pτ energy point of view, instead, suggests that doing the experiment with 1.17 eV (ν=0.282 PHz, λ=1064 nm, τ=3.55 fs) at fluence 587 $\frac{mJ}{{cm}^{2}}$, and 2.3375 eV (ν=0.564 PHz, λ=532 nm, τ=1.77 fs) at fluence 2350 $\frac{mJ}{{cm}^{2}}$ will produce the same result. On the other hand, increasing or decreasing fluence from 0.4 $\frac{mJ}{{cm}^{2}}$ in the 41 meV case would not trigger superconductivity.

A similar situation is described by M Mitrano *et al*. [18], who study photo-induced room temperature superconductivity in K_3_C_60_ as well. These authors compare the photoexcitation of this material from 50 K to room temperature with MIR light at 100 meV (ν=25.4 THz, λ=11.8 μm, τ=39.37 fs) and fluence 1.1 $\frac{mJ}{{cm}^{2}}$, and near infrared (NIR) light at 600 meV (ν=144.8 THz, λ=2.07 μm, τ=6.91 fs) also at fluence 1.1 $\frac{mJ}{{cm}^{2}}$. The excitation is provided by lasers at repetition rate which we assume to be the same as that in Ref. 20, i.e. R=100 MHz. With this assumption on R, we estimate the average power P_ave_ to be P_ave_=110 $\frac{kW}{{cm}^{2}}$. The energy Pτ is then 4.3 $\frac{nJ}{{cm}^{2}}$ for the 100 meV laser, and 0.76 $\frac{nJ}{{cm}^{2}}$ for the 600 meV one. Assuming l_act_=1 μm, for the 100 meV case P_λave_=1.3 $\frac{MW}{{cm}^{2}}$ and P_λ_τ=51.1 nJ. This excitation enables a reduction in σ_1_(ω), as reported in Fig. 4(d) of Ref. 21. On the other hand, for the 600 meV case, P_λave_=0.23 $\frac{MW}{{cm}^{2}}$, and P_λ_τ=1.6 nJ. This excitation does not produce a reduction in σ_1_(ω), as also reported in Fig. 4(d) of Ref. 21. So, we may safely conclude that the two orders of magnitude lower amount of energy transferred from the 600 meV laser hinders the reduction of σ_1_(ω). In contrast, however, M Mitrano *et al*. [21] ascribe the result with the lack of excitation of the IR-active T_1u_ phonon in K_3_C_60_ in the case of the 600 meV laser.

1. **Accuracy of Random Forest Classifier**

A random forest classifier was built to select the features that most distinguish the light exposure source in the video. 3018 features and 282 observations, split into training and testing data 70/30, were used to build the classifier. The resulting classifier had 100% accuracy on the training data and 89% accuracy on the withheld testing data. Confusion matrices for the testing data are shown below in S3 Table. Verification on an external dataset was not available, and because the goal was variable selection rather than classification, we did not feel this step was necessary.

**Table S3. Confusion matrix for the data on C. elegans.**

| Truth →  Prediction ↓ | Blue Light | Red Light | Radio Wave | White Light |
| --- | --- | --- | --- | --- |
| Blue Light | 4 | 0 | 1 | 0 |
| Red Light | 0 | 4 | 0 | 0 |
| Radio Wave | 1 | 0 | 55 | 5 |
| White Light | 0 | 0 | 2 | 12 |

Confusion matrix for the random forest classifier tested on withheld data (30% of observations).

**References**

1. Rybarczyk RJ, Federick AED, Kokhan O, Luckay R, Scarel G. Probing electromagnetic wave energy with an in-series assembly of thermoelectric devices. AIP Adv. 2022 Apr 1;12(4):045201.

2. Griffiths DJ. Introduction to electrodynamics. 4. ed., international ed. Boston: Pearson; 2013. 599 p. (Always learning).

3. Jackson JD. Classical electrodynamics. 3. ed., [Nachdr.]. Hoboken, NY: Wiley; 2009. 808 p.

4. Lozano C, Ten Hagen B, Löwen H, Bechinger C. Phototaxis of synthetic microswimmers in optical landscapes. Nat Commun. 2016 Sept 30;7(1):12828.

5. Jayalakshmi Y, Van Duijneveldt JS, Beysens D. Behavior of density and refractive index in mixtures of 2,6-lutidine and water. J Chem Phys. 1994 Jan 1;100(1):604–9.

6. Reimann R, Doderer M, Hebestreit E, Diehl R, Frimmer M, Windey D, et al. GHz Rotation of an Optically Trapped Nanoparticle in Vacuum. Phys Rev Lett. 2018 July 20;121(3):033602.

7. Van Der Laan F, Reimann R, Militaru A, Tebbenjohanns F, Windey D, Frimmer M, et al. Optically levitated rotor at its thermal limit of frequency stability. Phys Rev A. 2020 July 6;102(1):013505.

8. Van Der Laan F, Reimann R, Doderer M, Hebestreit E, Diehl R, Frimmer M, et al. Erratum: GHz Rotation of an Optically Trapped Nanoparticle in Vacuum [Phys. Rev. Lett. **121** , 033602 (2018)]. Phys Rev Lett. 2021 Apr 15;126(15):159901.

9. Ahn J, Xu Z, Bang J, Deng YH, Hoang TM, Han Q, et al. Optically Levitated Nanodumbbell Torsion Balance and GHz Nanomechanical Rotor. Phys Rev Lett. 2018 July 20;121(3):033603.

10. Reimann R, Private communication

11. Lian Z, Kobayashi Y, Vequizo JJM, Ranasinghe CSK, Yamakata A, Nagai T, et al. Harnessing infrared solar energy with plasmonic energy upconversion. Nat Sustain. 2022 Oct 17;5(12):1092–9.

12. Friese MEJ, Nieminen TA, Heckenberg NR, Rubinsztein-Dunlop H. Optical alignment and spinning of laser-trapped microscopic particles. Nature. 1998 July 23;394(6691):348–50.

13. Π=–{sin(kd(n_o_-n_e_))cos2φsin2θ} + {1-cos(kd(n_o_-n_e_))}sin2φ, where:

k=free-space wavenumber

d=thickness

n_o_, n_e_=refractive indices of the birefringent material experienced by the ordinary and extraordinary rays, respectively

φ=degree pf ellipticity of the light

θ=angle between the fast axis of the quarter-wave plate producing the elliptically polarized light and the optic axis of the birefringent material.

14. Kuhn S, Kosloff A, Stickler BA, Patolsky F, Hornberger K, Arndt M, et al. Full rotational control of levitated silicon nanorods. Optica. 2017 Mar 20;4(3):356.

15. Mous S, Gotthard G, Ehrenberg D, Sen S, Weinert T, Johnson PJM, et al. Dynamics and mechanism of a light-driven chloride pump. Science. 2022 Feb 25;375(6583):845–51.

16. Jardetzky O. Simple Allosteric Model for Membrane Pumps. Nature. 1966 Aug;211(5052):969–70.

17. Tsukamoto T, Yoshizawa S, Kikukawa T, Demura M, Sudo Y. Implications for the Light-Driven Chloride Ion Transport Mechanism of *Nonlabens marinus* Rhodopsin 3 by Its Photochemical Characteristics. J Phys Chem B. 2017 Mar 9;121(9):2027–38.

18. Christou NE, Apostolopoulou V, Melo DVM, Ruppert M, Fadini A, Henkel A, et al. Time-resolved crystallography captures light-driven DNA repair. Science. 2023 Dec;382(6674):1015–20.

19. Huang S, Shen L, Roelfsema MRG, Becker D, Hedrich R. Light-gated channelrhodopsin sparks proton-induced calcium release in guard cells. Science. 2023 Dec 15;382(6676):1314–8.

20. Rowe E, Yuan B, Buzzi M, Jotzu G, Zhu Y, Fechner M, et al. Resonant enhancement of photo-induced superconductivity in K3C60. Nat Phys. 2023 Dec;19(12):1821–6.

21. Mitrano M, Cantaluppi A, Nicoletti D, Kaiser S, Perucchi A, Lupi S, et al. Possible light-induced superconductivity in K3C60 at high temperature. Nature. 2016 Feb;530(7591):461–4.
